# Scale and context dependency of deforestation drivers: Insights from spatial econometrics in the tropics

**DOI:** 10.1371/journal.pone.0226830

**Published:** 2020-01-29

**Authors:** Rubén Ferrer Velasco, Margret Köthke, Melvin Lippe, Sven Günter

**Affiliations:** 1 Department of Ecology and Ecosystem Sciences, School of Life Sciences Weihenstephan, Technical University of Munich, Freising, Germany; 2 Institute of International Forestry and Forest Economics, Johann Heinrich von Thünen Institute, Hamburg, Germany; University of the Aegean School of Social Sciences, GREECE

## Abstract

A better understanding of deforestation drivers across countries and spatial scales is a precondition for designing efficient international policies and coherent land use planning strategies such as REDD+. However, it is so far unclear if the well-studied drivers of tropical deforestation behave similarly across nested subnational jurisdictions, which is crucial for efficient policy implementation. We selected three countries in Africa, America and Asia, which present very different tropical contexts. Making use of spatial econometrics and a multi-level approach, we conducted a set of regressions comprising 3,035 administrative units from the three countries at micro-level, plus 361 and 49 at meso- and macro-level, respectively. We included forest cover as dependent variable and seven physio-geographic and socioeconomic indicators of well-known drivers of deforestation as explanatory variables. With this, we could provide a first set of highly significant econometric models of pantropical deforestation that consider subnational units. We identified recurrent drivers across countries and scales, namely population pressure and the natural condition of land suitability for crop production. The impacts of demography on forest cover were strikingly strong across contexts, suggesting clear limitations of sectoral policy. Our findings also revealed scale and context dependencies, such as an increased heterogeneity at local scopes, with a higher and more diverse number of significant determinants of forest cover. Additionally, we detected stronger spatial interactions at smaller levels, providing empirical evidence that certain deforestation forces occur independently of the existing *de jure* governance boundaries. We demonstrated that neglecting spatial dependencies in this type of studies can lead to several misinterpretations. We therefore advocate, that the design and enforcement of policy instruments—such as REDD+—should start from common international entry points that ensure for coherent agricultural and demographic policies. In order to achieve a long-term impact on the ground, these policies need to have enough flexibility to be modified and adapted to specific national, regional or local conditions.

## 1 Introduction

Deforestation processes are related to human activities and endangering forest ecosystem services in many cases [1–3]. These impacts on e.g. carbon sequestration, soil and water quality, species losses or local livelihoods, are widely discussed among the scientific community [4,5]. The discussion is especially recurrent for the pantropics [6,7], where the FAO reported a net annual loss of forest area of about 7 million hectares for the 2000–2010 period [8]. More recent (2003–2014) average net carbon losses in tropical regions have been estimated in 452.2+92.0 Tg C · yr^-1^ globally, of which 59.8% are attributable to America, 23.8% to Africa, and 16.3% to Asia [9]. In parallel, the main drivers behind tropical forest cover (FC) change have been repeatedly studied as well [10–12]. In 2012, Hosonuma et al. [11] related over 80% of global deforestation for the 2000–2010 period to agricultural expansion (both commercial and subsistence), followed by other anthropogenic causes, namely mining, infrastructure and urban expansion. In a more recent study in 2018 [12], Curtis et al. quantified the global forest loss between 2001–2015 and attributed it to permanent land use changes due to commodity production (27%), forestry (26%), shifting agriculture (24%), and wildfire (23%). Busch and Ferretti-Gallon compiled in 2017 [13] “a comprehensive database of 121 spatially explicit econometric studies of deforestation published in peer-reviewed academic journals from 1996 to 2013”. In these studies, variables related to population, built infrastructure and market demand for agriculture were consistently associated with high deforestation, while poverty, higher elevations and steeper slopes were regularly identified with lower forest loss. Other variables related to aspects such as ownership and management rights, market demand for timber, or further socioeconomic and biophysical characteristics, showed varying or no influence on FC across the studies included in the meta-analysis.

Gathering knowledge about the drivers of deforestation across different jurisdictional levels is as a precondition for designing effective land use planning and policies at the levels where forest governance takes place. As an example of this, we can highlight the references for the design and implementation of operative and efficient strategies of REDD+ projects [14–16]. Moreover, generalizations about deforestation across pantropical regions and across different jurisdictional levels would help predicting future changes which might occur with or without policy interventions [17]. An area of research that already points in this direction is the classification of tropical countries or regions—and their drivers of deforestation—based on their FC levels and deforestation rates [11,17–19]. This is frequently made under the assumptions of the forest transition theory, which describes the existence of recurrent phases of FC decline and re-expansion [20,21].

Despite the number of studies dealing with the identification, categorization and quantification of the main drivers of FC change in the tropics, no empirical study of global or pantropical focus considered the behavior of these drivers in subnational administrative units across spatial scales and countries so far. On the one hand, some authors have conducted subnational or even multilevel approaches to analyze the causes of deforestation within different interrelated administrative hierarchies, but always putting their focus on single countries (e.g. [22–25]). On the other hand, supranational-regional and global studies have always focused on national and regional aggregations (e.g. [11,12,26]). For instance, from the 121 studies included in Busch and Ferretti-Gallon’s meta-analysis [13], only nine included *de jure* administrative entities as their units of observation, and only four studies analyzed data from different tropical regions [27–30]. However, no econometric study of pantropical scope has focused on the drivers of deforestation at sub-national jurisdictions so far.

In order to address this research gap, we made use of spatial econometrics and conducted a multi-level approach with nested jurisdictional units in three tropical contexts of Africa, Asia and South America. Spatial econometrics is a discipline with increasing interest in urban and regional studies [31–34], which can contribute to a better understanding of spatial phenomena and tropical deforestation patterns at different interconnected subnational administrative levels. For instance, with the use of local spatial models, it is possible to estimate the spillovers and the indirect impacts of neighboring units [35]. Furthermore, these models can provide information about omitted variables and on how spatial clusters look like [33,36,37]. So far, these methods have not been widely used in previous studies of tropical deforestation, even if local interactions between neighbor administrative units and omitted spatially correlated parameters exist in real physical deforestation processes. Again, from all the spatially explicit econometric studies included in Busch and Ferretti-Gallon’s meta-analysis [13], more than the half of them did not even report any treatment of spatial autocorrelation. Furthermore, only seven of the remaining studies (5.8% of the total) considered spatial lags or the use of a weighting neighbor matrix, but always focusing on one single country or region and at one single level of analysis (e.g. [38,39]). This was also the case in more recently published studies (for instance: [39,40]).

Within this context, our study wants to address the following research questions: Are well-studied *global drivers of tropical deforestation* also *constant across* different subnational *administrative levels*? If not, which *differences* are observable and at which jurisdictional levels? Is this the same for different *tropical contexts*, or are there country/region specific behaviors?

## 2 Materials and methods

### 2.1 Selection of study areas and the forest transition theory

With the selection of the study areas we aimed to include three countries that accounted for as much pantropical variability as possible, regarding their FC and deforestation rates, but also considering their biophysical, geographical, socioeconomic and demographic conditions. A key factor behind this selection process was the situation of each country within the forest transition curve [20], when observed at national scale (see S1 Fig). Based on this, we selected the three following countries:

1. Zambia is a land-locked plateau in south-central Africa, which in 2010 was still in the pre-/early stage of the forest transition [11] with a high FC (65.4%) and moderate deforestation rates (-0.3% · yr^-1^) [41]. Zambia has relatively low population density, life expectancy at birth, GDP per capita and HDI [42–44]. According to Global Forest Watch, the deforestation rates in Zambia have increased and accelerated significantly in the last ten years [1]. While the country lost 850 kha of tree cover extent with canopy larger than 10% during the period 2001–2009, this loss more than doubled to 1.96 Mha in the period 2010–2018. This accelerated deforestation might indicate that Zambia already entered its early transition, reducing its total FC to 62% in 2015 [45]. Following Curtis et al. [12], most of this deforestation was due to shifting agriculture. Other identified relevant drivers of deforestation (and degradation) in Zambia are mining and infrastructure development, wood extraction, charcoal production and wild fires [46].
2. Ecuador is a mega-diversity hotspot that shelters the Andes and the Amazon basin, in the Pacific side of northwestern South America. Ecuador has reduced FC to about 50%, but deforestation is still ongoing since the late nineties at relatively high rates (-0.6% · yr^-1^) [41,47]. The forest context in the country can thus be a clear example of a “frontier area” ([17]). Ecuador has twice the population density of Zambia, with a share of 63% of urban population, and a relatively high GDP and HDI [42–44]. The key driver of deforestation in Ecuador is again shifting agriculture [12], together with small-scale ranching and, in a more local manner, commodity-production such as palm oil [48].
3. The Philippines is an archipelago in Southeast Asia consisting of over 7,000 islands. This country is supposed to have achieved a net FC increase of 0.8% · yr^-1^ between 1990 and 2015, with less than 30% of FC left in 2015 [41]. The Philippines is very densely populated, exhibits the highest road density among the three countries, and a share of 41% of agricultural land [42–44]. According to Global Forest Watch and Curtis et al. [1,12], tree cover loss in the Philippines is mostly commodity-driven and related to agriculture expansion. Forestry practices and urbanization also play a bigger role on deforestation than in Zambia and Ecuador. The forest situation on the Philippines is thus an example of a clear “forest-agricultural mosaic” or late-post forest transition phase, when observed at national level [11,17].

### 2.2 Units of observation and levels of analysis

**Fig 1:**
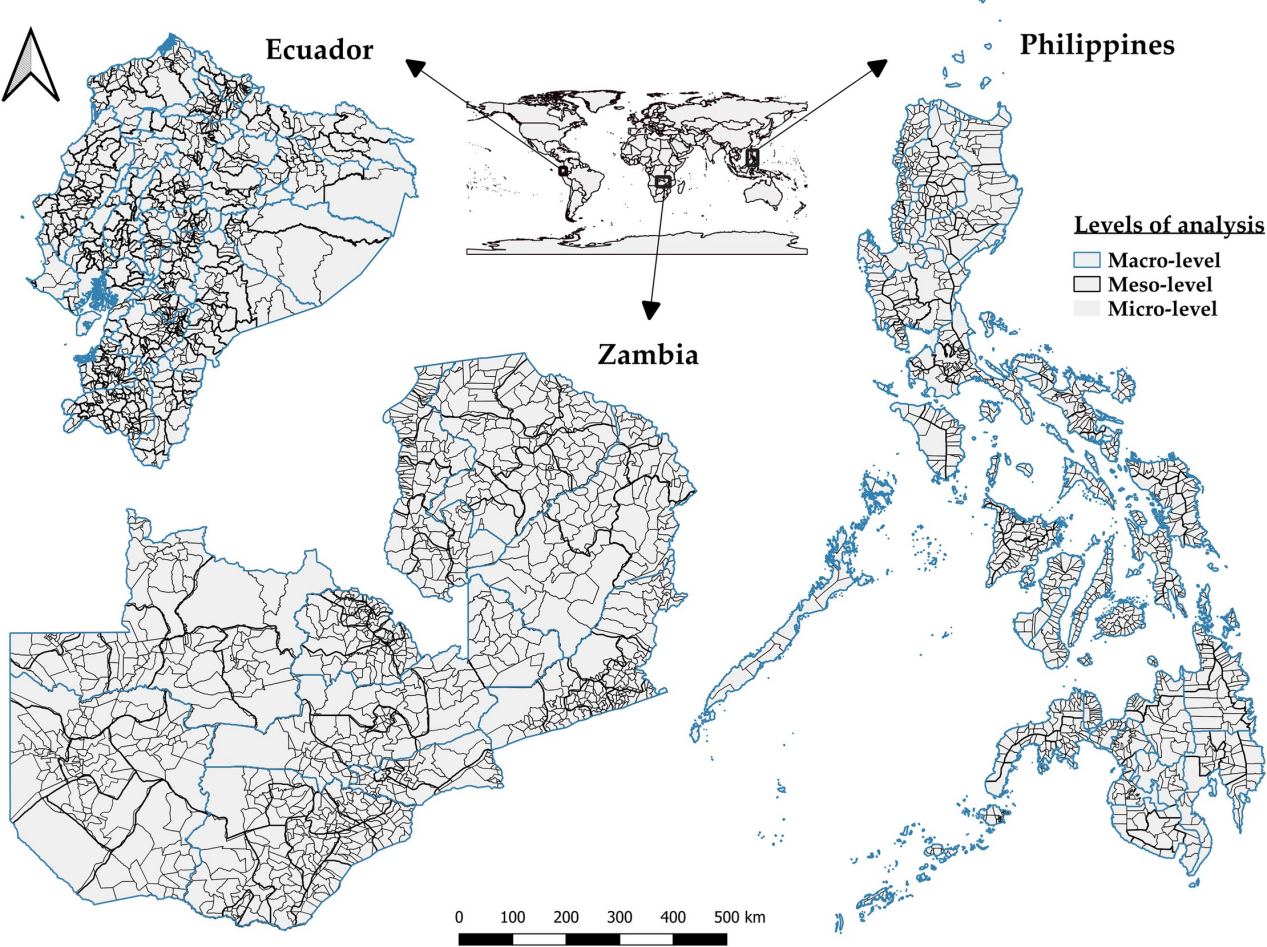
Maps of the three selected countries and their corresponding jurisdictional/spatial levels of analysis. The countries are displayed at the same scale with proportional sizes.

We subdivided each of the selected countries into three nested spatial levels of analysis (macro-, meso-, and micro-level), related to their hierarchical legal administrative configuration (Fig 1). Each of these three levels of analysis corresponds to an existing *de jure* governance structure, with comparable competences regarding forest policy design and implementation across the three countries [49,50].

For Zambia, 9 provinces comprise the macro-level and 71 districts the meso-level. We downloaded the geo-referenced data for both levels from the GADM database [51]. The third level (micro-) represents approximations of 1,358 ward and constituency boundaries based on printed information from the Election Commission of Zambia (ECZ) for which a polygon file produced by Eubank (2014) [52] was used. The main institution responsible for the management of forest resources in Zambia is the Forestry Department of the Ministry of Lands, Natural Resources and Environmental Protection (MLNREP). Zambia is experiencing a national decentralization process which aims to increase the power and obligations of the districts (meso-level) in order to improve the quality of the service delivery at the subnational level [53–56]. In this line, some changes have happened in the last decade regarding the national legal framework for the forestry sector, like the inclusion of local forest regulations and other forms of local forest management: e.g. Joint Forest Management (JFM) or community forestry [57–60].

In the case of Ecuador, the administrative units selected for the macro-level are the 24 provinces plus the three non-delineated zones as one single unit. The meso-level includes 224 counties and the micro-level 1,024 parishes. We downloaded the data regarding these boundaries from the National Institute of Statistics and Census database [51,61]. Although the main actions regarding forest policy and management in Ecuador are basically planed and coordinated at national level by the Ministry of Environment (MAE) [62], these three levels of territorial organization (provinces, counties, parishes), whose legislative-political role is acknowledged by the current Ecuadorian Constitution [63] and the Organic Code of Territorial Organization, Autonomy and Decentralization [64], participate actively in the implementation of MAEs policies or other forest management programs in line with the national laws.

For the Philippines, the three jurisdictional levels of analysis include 17 regions (macro-level), 81 provinces (meso-level) and 1,652 municipalities (micro-level). The geographic datasets were extracted from the GADM database [51] and are based on official boundaries from NAMRIA (National Mapping and Resource Information Authority), which can be acquired at the Philippine Geoportal System [65]. At a national level, the main governmental body which deals with forest management planning in the Philippines is the Forest Management Bureau (FMB), which belongs to the Department of Environment and Natural Resources (DENR). DENR has offices in all the administrative regions (macro-level), and some offices that operate in most of the provinces (PENROs, at meso-level) and in some cities or municipalities (CENROs, at micro-level) [66,67].

### 2.3 Selection of variables: Building of a spatial database

We built a geodatabase with the support of Geographic Information System (GIS) software and tools: QGIS 3.4. [68]. This geodatabase (see S1 File) comprised the downloaded spatially explicit boundaries for the three jurisdictional levels in the three countries. In the next steps, we included the information about FC and relevant drivers of de- and reforestation (response and explanatory variables) for each of these units and levels of analysis, as described in the following subchapters.

#### 2.3.1 Response variable: Forest cover (FC)

We extracted the FC information for each administrative unit (three countries and three levels) from the most recent national land cover map that was available at the time of performing this study [47,69,70] (Table 1). Therefore, we conducted a cross-sectional analysis, which assumed that patterns of FC development can be detached from the temporal scale, as they are dependent of socioeconomic development [20,21,71]. National land cover maps, contrary to global datasets like [1,72,73], presented advantages such as higher resolution and accuracies for the regions of interest, while considering particular land cover characteristics of each tropical context.

**Table 1:** Land cover map sources and FC classification used in this study.

Country:		Zambia	Ecuador	Philippines
Year and source:		2016, [69]	2014, [47]	2010, [70]
Sensor(s) Used:		Sentinel-2	LandSat, RapidEye	ALOS AVNIR-2, SPOT 5, LandSat
Resolution:		~20m	~5-30m	~10-30m
**Potential forest area** (**FA**_**pot**_^**2**^**)**	**Forest area** **(FA**^**1**^**)**	Tree Cover Areas	Native forest	Closed forest
			Forest plantations	Open forest
				Mangrove forest
	**Other** **non-forest vegetation area** **(FA**_**pot**_**−FA)**	Shrub cover areas	Herbaceous vegetation	Wooded grassland
		Grassland	Shrub vegetation	Grassland
		Cropland	Pasture	Shrubs
		Vegetation aquatic	Agricultural mosaic	Perennial crop
		Lichens	Permanent crop	Annual crop
		Sparse vegetation	Semi-permanent crop	Fallow
			Annual crop	
**Non-potential forest area** (**Non-FApot)**		Páramo	Bare areas	Open barren
		Natural (rivers)	Built-up	Marshland
		Infrastructure	Snow or ice	Swamp
		Glacier	Open water	Inland water
		Artificial		Fishpond
		Non-vegetation cover		Built-up
		Settlement		

^1^ FA: Forest area [ha]

^2^ FApot: Potential forest area [ha]

Thus, we post-classified and harmonized forest and land cover definitions, by aggregation of existing tree and FC classes into only three major land cover types: Forest area (FA), Potential forest vegetation area (FApot) and Non-potential forest vegetation area (Non-FApot). We obtained the dependent variable FC by normalizing the forest area (FA) as a fraction of a unit’s potential forest area (FApot) (Table 2). FC is, therefore, a proportion of each jurisdictional unit’s total forest area on its potentially forested area, rather than on its total surface. The concept of potentially forested area as interpreted in this study is aiming to estimate the maximum forest area that could be reached in a limited period of time, similar to the approach of Köthke et al. (2013) [26]. We calculated this by aggregating all relevant vegetation land cover types, while classes not suitable for forest vegetation were consequently excluded (e.g. water bodies, glaciers or bare areas). Built-up and artificial infrastructures were neither included into this aggregate, assuming that urban areas are rather unlikely to experience rapid land cover dynamics [74,75]. This allows the range for the dependent variable to vary between 0 and 100%.

**Table 2:** Variables considered in the study (in bold) and related definitions and sources.

	Definition and [unit]	Sources	Year(s) / Country			
*Dependent variable*			**ZAM**	**ECU**	**PHI**	
**FC**	**Forest Cover [%]**	FA/FA_pot_^1^	2016	2014	2010	
	FA: total forest area [ha]	[47,69,70] ^1^	2016	2014	2010	
	FA_pot_: Potential forest area [ha]	[47,69,70] ^1^	2016	2014	2010	
*Explanatory variables*			**ZAM**	**ECU**	**PHI**	**Expected impact**
**A**_**TOT**_	**Total Area [ha]**	[51,52,61]	2006–10	2010	2010	Positive
**PVA**	**Share of potential vegetation on surface area [%]**	[FA_pot_/A_TOT_] ^1^	2016	2014	2010	Negative
**PP**_**FA**_	**Population pressure on remaining forest area [pers./ha]**	[P_TOT_ /A_TOT_]	2015–16	2014–15	2010	Negative
	POP_TOT_: Total population [pers.]	[76–79]	2015	2015	2010	
**RD**	**Road density [km/km**^**2**^**]**	[RTOT/A_TOT_]	2016	2016	2016	Negative
	R_TOT_: Total road length [km]	[80]	2016	2016	2016	
**FL**	**Flatness: share of surface with less than 16% steepness [%]**	[FL_TOT_/A_TOT_]	2008	2008	2008	Negative
	FL_TOT_: Total area with low slopes (<16%) [ha]	[81]	2008	2008	2008	
**CSI**	**Crop suitability index [%]**	[82,83]	2005	2005	2005	Negative
**CY**	**Maximum cereal area yield [kcal/ha]**	[84–86]	2005–15	2004–14	2000–10	Positive

^1^ From Table 1

#### 2.3.2 Explanatory variables: Drivers of forest cover change

We selected seven explanatory variables, which included elements related to physio-geographic, demographic and socio-economic aspects that we expected to influence FC (Table 2). These variables have been often identified and discussed as mostly influential and uniform across tropical countries by previous authors (see, for instance, [10,13]).

We tested the influence of total area (A_TOT_), as different sizes of administrative units might be subject of differences in land pressure, political processes, as well as options for trade and cooperation [26]. We extracted the total extent of each administrative unit from the spatial boundaries used to define the study’s units of analysis. We did every calculation of areas or slope degrees (see parameter FL) after re-converting the source spatial files into the corresponding Universal Transverse Mercator (UTM) projected coordinate system. We used UTM zones 35S, 17S and 51N for Zambia, Ecuador and Philippines respectively.

The potential vegetation area (PVA) describes the share of potentially forested area (FA_POT_) related to the total area (A_TOT_) of the analyzed administrative unit. PVA can range from 0 to 100% and high values signify a potential for higher forest area in the unit, but not necessarily its existence [26]. For instance, a large region in the Amazon with a lot of its surface share covered by native forest would rank high in PVA. At the same time, a smaller region in a rural province, which has been deforested centuries ago and nowadays mostly comprises pasture- and croplands, would also rank high in PVA. Therefore, and as FC in this study is defined as a proportion on PVA, high PVA values are expected to decrease FC. Units with high PVA will in general have more options to establish productive locations for agricultural land and they are expected to experience an increased need to exploit for food production within the region’s borders.

A key driver of deforestation is the role of population density and demographic development [13,87,88]. We expect that higher population density result in higher demand for land and resources with related phenomena putting direct pressure on forest itself, like e.g. agricultural expansion and shifting cultivation, establishment of settlements, roads and other infrastructures, fuel wood collection and resource extraction. We estimated the total population (P_TOT_) for each administrative unit by extracting the associated demographic data from worldpop.org.uk [76–79]. We did this for the year closest to the corresponding land cover map used in each country (Table 2). We calculated the density (pressure) on the remaining total forest area (PP_FA_) by dividing the total population by the forest area (FA).

Similar to population pressure, indicators for accessibility, such as road density or distance to roads, have been widely used as a measure of environmental pressure and economic development [89–92]. The presence of roads can contribute to a range of pressures on forests and on the natural environment in general [13] and thus, we expect that high road densities are likely to affect FC negatively. We downloaded and calculated the total road length (R_TOT_) in each assessed jurisdictional unit (including highways, roads, paths and railways) from openstreetmap.org and geofabrik.de [80]. The total road length in km (R_TOT_) was divided by the total area (A_TOT_) of each respective administrative unit (in km^2^), to calculate road density (RD).

Slope at 90m resolution was calculated from the 4.1 version of the SRTM DEM (Shuttle Radar Topographic Mission Digital Elevation Model) produced by the NASA (National Aeronautics and Space Administration) and CGIAR (Consultative Group for International Agricultural Research) [81]. For each analyzed administrative unit, we divided the total area below 16% slope (FL_TOT_) by its total area (A_TOT_), thus generating a flatness indicator: FL. We selected land under 16% steepness based on the FAO definition of non- (0–8%) or slightly (8–16%) constrained rain-fed land ([82]). We therefore expect, that regions with a higher share of flatness are more suitable for the clearing of new agricultural land have a lower FC [13]. Cross-country and cross-level differences are also expected depending on each specific physio-geographic condition.

We estimated the crop suitability index (CSI) from FAO’s FGGD (Food Insecurity, Poverty and Environment Global GIS Database) data regarding ‘suitability of currently available land area for rain-fed crops, using maximizing crop and technology mix’ [82,83]. This dataset is a global raster layer displaying values between 0 (not suitable) and 100 (very high CSI). We gave a zero value (no crop suitability) to classes like internal water bodies, urban, closed forest, protected areas, or irrigated land. This concerned a few specific areas like the Galapagos, remote Amazonian forest, or the metropole of Manila in the Philippines. As the pixel resolution was rather coarse (1/12 of degree)—especially when considering the size of some units from the smallest jurisdictional level -, we calculated the area-weighted mean of pixel values situated within the boundaries for each unit of analysis. CSI represents the agricultural potential of the land and is, thus, expected to affect FC negatively [13].

Finally, the cereal area yield (CY) expresses the actually achieved yield at a point or period of time. The CY is supposed to increase over time, fluctuate short-term and maybe saturate in a stage of high intensification. The cereal area yield of a region is an indicator of agricultural productivity and intensification [93,94]. Thus, we expect it to release pressure on FC. We selected two main cereal categories, which represent major crop types in the three selected countries, namely maize and rice. We obtained data for aggregated maize and rice classes area production yields (MY, RY in tons/ha) from official national sources between 1987 and 2015 [84–86], for five of the twelve analyzed samples. For both cereal types, we considered the arithmetic mean of the last 10 years before the production of each particular land cover. We converted the computed means to caloric yields (kcal/ha), using the general conversion factors presented by Cassidy et al. (2013) [95]. For those administrative units with information for both maize and rice, the highest caloric yield was taken into consideration, assuming that this crop type is more likely occurring in the respective region.

### 2.4 Spatial econometric modelling

We conducted the spatial econometric analysis with the support of the JMP^®^ 13.1.0 and R 3.5.2 statistical software [96,97] and the *spdep* [98,99], *rgdal* [100], *sp* [101], *rgeos* [102] and *RANN* [103] packages. The final product and files used for the analysis (S1 File), together with the associated R script (S2 File), can be found in the online attachments of this article. We defined a total of twelve samples: nine samples present the combinations of the three countries and the three spatial levels of analysis, and three samples present the aggregated data (pantropical) of all countries at the three spatial levels.

We assumed a sigmoidal relationship between the drivers of deforestation and the dependent variable, following the results of other authors [26,88,104,105]. This relationship constitutes a model of FC decline with an inverted growth function approaching 1 as horizontal asymptote at the left side and 0 at the right side (similar to the graph as shown in S1 Fig). Subsequently, the dependent variable needed to be linearized by logistic transformation in order to permit linear regression techniques and the explanatory variables were transformed using a logarithm function:

$${\mathrm{FC}}_{s}^{*}=\ln\left(\frac{1}{{\mathrm{FC}}_{s}}‐1\right)=\ln\left(\frac{FA_{{\mathrm{POT}}_{s}}}{{\mathrm{FA}}_{s}}‐1\right)$$ (Eq 1)

$$X_{v,s}^{*}=\ln\;X_{v,s}$$ (Eq 2)

where: * refers to linearized or transformed; *s* is the sample; and *X* is a vector of the seven explanatory variables *v*.

The samples with missing, not linearizable extreme or nil values–in the case of FC, PVA, CSI or RD–were dismissed. This generally consisted of micro- or meso-units from either (a) metropoles with no registered FC (mainly a few big urban centers in Copperbelt and Lusaka in Zambia, highly populated cities in the Philippines and a small number of settlements belonging to the arid Andes, Quito or Guayaquil in Ecuador), or (b) remote areas with almost inexistent human presence (like the Galapagos in Ecuador or the Turtle Islands in Philippines). This implied the exclusion of a total 3.92% of the macro-units, 3.99% of the meso-units and 24.67% of the micro-units from the original sample.

For each of the twelve samples, the provided explanatory variables were standardized individually as follows, in order to later compare or estimate their relative contribution to the model:

Xv,s^=Xv,s*‐μ(Xv,s*)σ(Xv,s*)=lnXv,s‐μ(lnXv,s)σ(lnXv,s) (Eq 3)

where: *X*^*^*^_*v*,*s*_ is the standardized explanatory variable *v* for sample *s*; μ(X^*^_v,*s*_*)* represents the mean value in sample *s* for the transformed explanatory variable *v*; and σ(X^*^_v,*s*_*)* is the standard deviation of the transformed explanatory variable *v* in sample *s*.

In a next step, we tested collinearity between the seven explanatory variables for every sample. Variables with bivariate correlation values of at least 0.6 were considered as highly correlated predictors. We performed simple linear regressions for each of the independent variables. The highly correlated variables with the lower coefficients of determination in their respective linear regressions were not included into the further calculations, assuming they were providing redundant information. Then, we identified the significant explanatory variables per sample, using the non-spatial OLS model following automated stepwise backwards elimination method with the smallest Bayesian information criterion as stop rule. Therefore, the OLS model for multivariate analysis is expressed like:

OLSModel:FCs*=β0+βvlnX^v,s+εs (Eq 4)

where *β* are the respective coefficients and *ε* is the residual.

**Fig 2:**
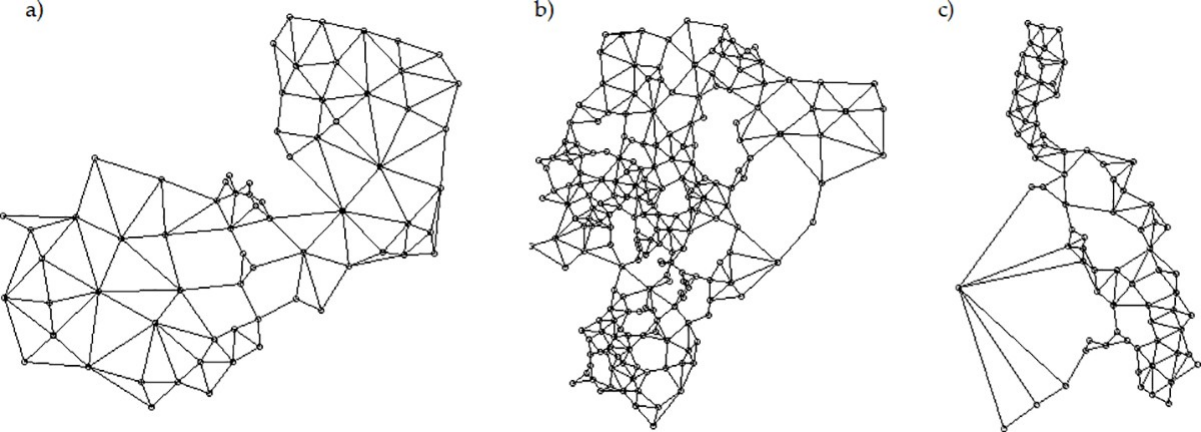
The SOI W diagrams representing the spatial interactions between the meso-level jurisdictional units. a) Zambia b) Ecuador and c) Philippines.

Next, we developed a spatial weights matrix (*W*) for each of the twelve samples. In order to avoid model deficiencies and misapplying spatial econometrics [106–108], this matrix should reflect how spatial units interact with each other and their degree of connectivity. We considered a graph-based—sphere of influence (SOI)—neighbor matrix for the twelve samples of our study (Fig 2). A SOI matrix works “based on Euclidean distances between polygon centroids, where points are neighbors if circles centered on the points, of radius equal to the points’ nearest neighbor distances, intersect in two places” [98,109].

We examined the results from each OLS analysis [98] to check for spatial dependency of the model residuals, by performing both Moran test [110] and to explore spatial relationships with the Lagrange Multiplier diagnostic for lag and error models [33,111,112]. We did this in order to reveal spatial autocorrelations and justify the use of the proposed econometric models. Thus, with this, we did not want to explore or discuss the spatial distributions of errors/variables explicitly for each context or scale, but we rather wanted to demonstrate the existence of spatial dependencies among the different samples (different contexts/scales) and justify the use of our spatial econometric models.

Next, to select the most suitable regression model for each sample, we applied the LeSage and Pace method [32,36] for local model specification. Thus, we did likelihood ratio (LHR) tests to select the spatial model that better explained each of the twelve samples. This method tries to demonstrate if a Spatial Durbin Error Model (SDEM) can be restricted to a simpler nested model, such as a spatial error model (SEM), a spatially-lagged X model (SLX), or reduced to the non-spatial OLS model:

Spatial Durbin Error Model (SDEM):

FCs*=β0+βvlnX^v,s+Ws,nθv,slnX^v,s,n+us,us=λWs,nus+εs (Eq 5)

if *θ* = 0, (6) results in Spatial Error Model (SEM):

FCs*=β0+βvlnX^v,s+us,us=λWs,nus+εs (Eq 6)

if *λ* = 0, (6) results in Spatially Lagged X Model (SLX):

FCs*=β0+βvlnX^v,s+Ws,nθv,slnX^v,s+εs (Eq 7)

if both *θ* = 0 and *λ* = 0, (6) results in OLS Model:

FCs*=β0+βvlnX^v,s+εs (Eq 8)

where: *W*_*s*,*n*_ represents the row-standardized weight of the neighbor *n* for a certain sample *s; θ*_*v*,*s*_ are the neighbors’ impacts on a certain variable *v* and sample *s*; *ln X*^*^*^_*v*,*s*,*n*_ represents the neighbors’ values for a certain variable and sample; *λW*_*s*,*n*_*u*_*s*_ represents the weighted spatial residual error.

**Fig 3:**
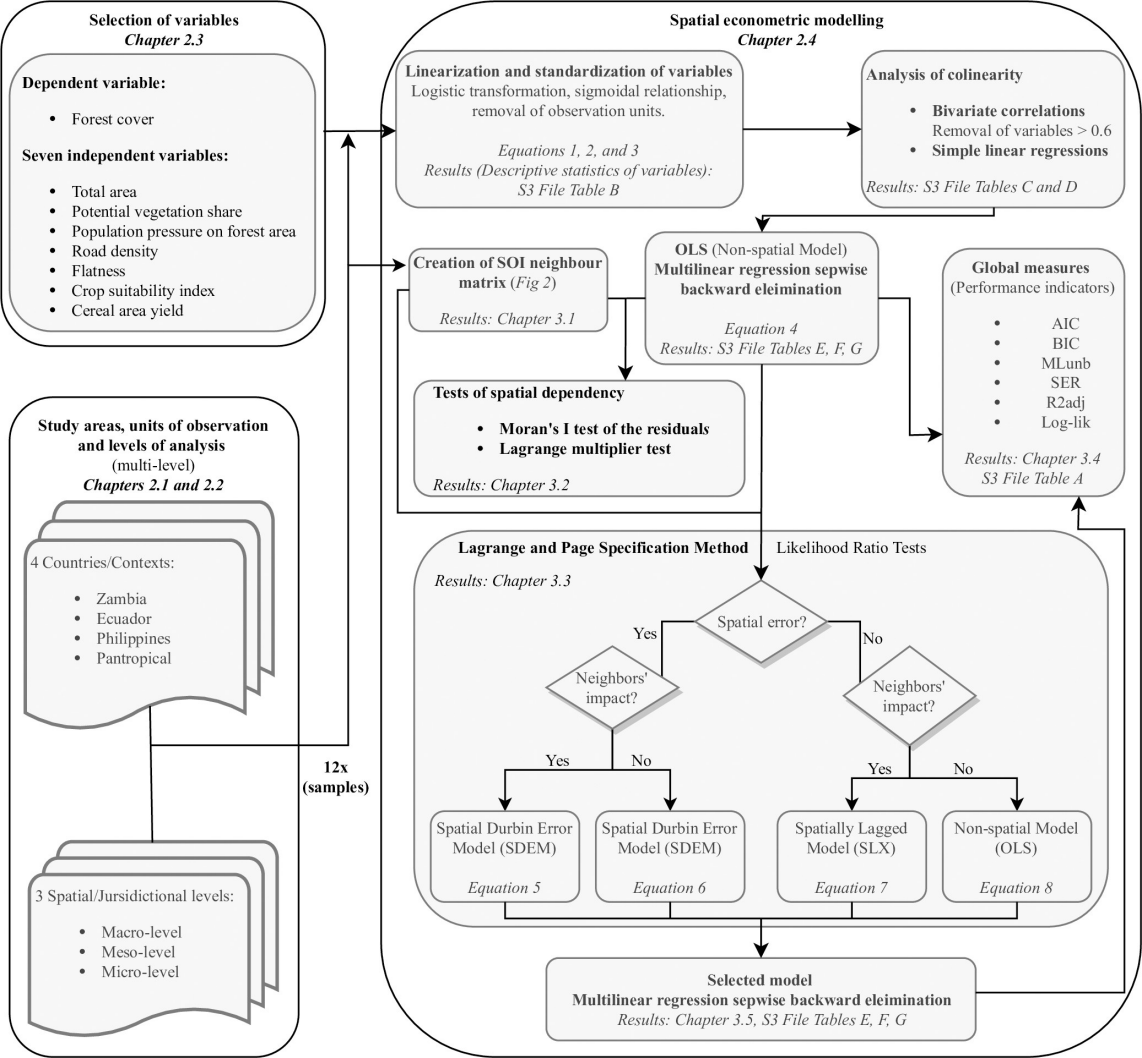
Conceptual diagram summarizing the analytical framework of this research article.

Thus, we assigned each sample to an optimal regression model, which could account for either neighbor impacts (SLX model), spatially correlated errors (SEM model), both spatial effects (SDEM model) or none of them (OLS model). Fig 3 summarizes the analytical framework of this research article in the form of a conceptual diagram. This graph also summarizes how the spatial interactions and the different proposed models refer to each other in the specification method.

Finally, in order to justify the specification method, we quantified and compared different performance indicators or global measures for both the OLS and the specified spatial models. First, we considered the (1) Akaike and (2) Bayesian information criterions (AIC, BIC). These are both estimators of relative quality of statistical models, where lower values indicate a better goodness of fit. We also calculated (3) unbiased maximum likelihood estimators of the error variance and (4) standard errors of regression. These two other measures estimate the goodness of fit in percentage, and they tend to decrease (approach zero) if the quality of the regression increases. Finally, we calculated (5) adjusted coefficients of determination (which indicate the percentage of the dependent variable variance explained by the model) and (6) log-likelihoods with maximum likelihood estimators for the regression coefficients. These two last parameters increase with improved model quality. All these measures were calculated following the formulas and definitions proposed by [113].

## 3 Results

### 3.1 Spatial weight matrices

Looking at the histograms of the number of neighboring regions (Table 3), we can observe similar distributions across the samples. The smallest matrix consists of 30 links, while the most complex one has 12,890 connections between regions. The matrices provide a relatively low number of sparse non-zero weight connections, especially in the pantropical model and at the smaller levels. These values range from 0.14% to 37.04%, which allowed us to perform the further spatial tests. The associated average links per matrix range from 3.3 (Zambia’s macro-level) to 4.6 (Ecuador’s micro-level) relations per sample [108].

**Table 3:** Summary of the applied SOI spatial weights matrix (*W*) for each sample [98].

Country	Level	Number of neighboring regions^1^									N^2^	N links^2^	Avg. links^2^	% NZW^2^
		1	2	3	4	5	6	7	8	9				
**PAN**^**3**^	Macro-	1	7	13	14	12	0	2	0	0	49	184	3.8	7.66
	Meso-	0	24	75	101	97	48	12	4	0	361	1,566	4.3	1.2
	Micro-	50	250	629	824	711	421	128	18	4	3,035	12,890	4.3	0.14
**ZAM**^**3**^	Macro-	0	2	3	3	1	0	0	0	0	9	30	3.3	37.04
	Meso-	0	3	18	16	16	12	2	3	0	70	314	4.5	6.41
	Micro-	7	45	172	287	272	174	49	8	2	1,016	4,590	4.5	0.44
**ECU**^**3**^	Macro-	1	4	4	6	7	0	2	0	0	24	94	3.9	16.32
	Meso-	0	15	39	58	60	31	7	2	0	212	930	4.4	2.07
	Micro-	5	35	130	223	264	155	46	6	1	865	3,986	4.6	0.53
**PHI**^**3**^	Macro-	0	1	6	5	4	0	0	0	0	16	60	3.8	23.44
	Meso-	0	6	18	26	20	7	2	0	0	79	326	4.1	5.22
	Micro-	39	168	331	309	184	87	30	6	0	1,154	4,304	3.7	0.32

^1^ Number of units with a certain number of neighboring regions (1–9).

^2^ N: Total sample size; N links: Total number of links per matrix (W); Avg. links: Average number of links per spatial unit in each matrix (W); % NZW: Percentage of links with non-zero weights in the matrix W.

^3^ PAN: Pantropical; ZAM: Zambia; ECU: Ecuador; PHI: Philippines.

### 3.2 Moran’s *I* and Lagrange multiplier tests

The Moran’s *I* test for the OLS residues was significant (considering a 1% threshold) in at least eight of the twelve samples (Table 4). We detected positive Moran’s *I* between 0 and 1 in these samples. At smaller administrative levels with bigger samples, *I* increases together with its standard deviation. Simultaneously, *I*s expected value and variance get closer to 0. Significances also reflect an increase in the tests with the smaller jurisdictional units within each country-specific sample. The aggregated pantropical models had the highest *I* values ranging from 0.41 to 0.58, just like the model for Zambia at the micro-level.

**Table 4:** Results of the Moran’s *I* and Lagrange multiplier tests from the OLS models.

			Moran test of the residuals (normal approximation) [110] Alternative hypothesis, greater. ^1^					Lagrange Multiplier test [111] ^2^							
								SEM		R-SEM		SLX		R-SLX	
Coun try^4^	Level	N	I	Exp.	Var.	SD	p-val^3^	LM	p-val^3^	LM	p-val^3^	LM	p-val^3^	LM	p-val^3^
**PAN**	**Macro-**	49	0.41	-3.95E-2	1.04E-2	4.40	***	14.29	***	16.14	***	0.58	n.s.	2.42	n.s.
	**Meso-**	361	0.52	-8.96E-3	1.31E-3	14.74	***	203.44	***	174.25	***	37.12	***	7.93	**
	**Micro-**	3,035	0.58	-1.12E-3	1.71E-4	44.55	***	1,970.20	***	1,753.80	***	355.64	***	139.32	***
**ZAM**	**Macro-**	9	-0.18	-1.66E-1	3.84E-2	-0.09	n.s.	0.48	n.s.	0.08	n.s.	2.71	.	2.30	n.s.
	**Meso-**	70	0.23	-3.06E-2	6.05E-3	3.41	***	8.13	**	4.00	*	4.19	*	0.06	n.s.
	**Micro-**	1,016	0.58	-3.11E-3	4.63E-4	27.09	***	718.80	***	272.23	***	446.61	***	0.04	n.s.
**ECU**	**Macro-**	24	0.13	-8.47E-2	1.71E-2	1.64	.	0.71	n.s.	0.86	n.s.	0.02	n.s.	0.17	n.s.
	**Meso-**	212	0.29	-1.47E-2	2.17E-3	6.58	***	37.31	***	23.80	***	15.16	***	1.64	n.s.
	**Micro-**	865	0.35	-3.97E-3	5.28E-4	15.49	***	231.56	***	135.61	***	105.66	***	9.72	**
**PHI**	**Macro-**	16	-0.15	-7.53E-2	2.69E-2	-0.48	n.s.	0.68	n.s.	0.88	n.s.	0.03	n.s.	0.92	n.s.
	**Meso-**	79	0.10	-2.27E-2	5.88E-3	1.59	.	1.54	n.s.	2.87	.	0.40	n.s.	1.73	n.s.
	**Micro-**	1,154	0.40	-2.82E-3	5.21E-4	17.48	***	299.24	***	259.71	***	39.54	***	0.01	n.s.

^1^ I: Moran’s *I*; Exp.: Moran’s *I* expected value under null hypothesis; Var.: *I* variance; SD: *I* Standard Deviate.

^2^ LM: Lagrange Multiplier Test; R-: Robust LM Test; SEM: Spatial Error Model; SLX: Spatially Lagged X Model.

^3^ p-val (p-values)

***: <10^−3^

**: <10^−2^

*: <5.10^−2^;.: <10^−1^; n.s.: >10^−1.^

^4^ PAN: Pantropical; ZAM: Zambia; ECU: Ecuador; PHI: Philippines.

The Lagrange multiplier (LM) tests (Table 4) reported strongly significant results (considering a 1% threshold) for eight of the twelve samples as well. The eight samples showed significant coefficients for the error model (SEM) test. Six of the samples also described significant results for the lagged X model (SLX). Moreover, seven and three samples were also significant at the robust tests for SEM and SLX, respectively. Significance for both error (SEM) and lagged-X (SLX) models increased at smaller administrative units in the different country samples. The significant values of LM for error models were always higher than those for lagged-X models in all of the studied samples, for both normal and robust tests. More specifically, for both Moran’s *I* and Lagrange Multiplier tests, the non-significant models were those of the macro-level in individual countries, with the smallest sample size, but also the model for the meso-level in the Philippines.

### 3.3 Model specification

We could specify a spatial model in nine of the twelve samples following the LeSage and Pace method. Table 5 shows the results of likelihood-ratio tests for the reduction of complex nested models, as described in Eqs 5 to 8.

**Table 5:** Results of the spatial model specification, following the LeSage & Pace [32,36] method by Likelihood Ratios (LHR) and nested model restriction.

			SEM^1^		SLX^1^		OLS^1^		
Country^3^	Level	N	LHR	p-val^2^	LHR	p-val^2^	LHR	p-val^2^	Selected Spatial Model ^1^
**PAN**	**Macro-**	49	0.56	n.s.	10.21	*	12.71	**	SEM
	**Meso-**	361	3.02	n.s.	172.50	***	188.98	***	SEM
	**Micro-**	3,035	133.63	***	1,804.10	***	2,000.90	***	SDEM
**ZAM**	**Macro-**	9	1.70	n.s.	0.08	n.s.	2.78	n.s.	None (OLS)
	**Meso-**	70	0.12	n.s.	7.36	**	7.90	*	SEM
	**Micro-**	1,016	37.59	***	686.60	***	808.74	***	SDEM
**ECU**	**Macro-**	24	5.41	n.s.	0.22	n.s.	6.04	n.s.	None (OLS)
	**Meso-**	212	10.82	*	36.63	***	48.89	***	SDEM
	**Micro-**	865	36.31	***	199.33	***	226.20	***	SDEM
**PHI**	**Macro-**	16	1.59	n.s.	0.73	n.s.	2.69	n.s.	None (OLS)
	**Meso-**	79	12.73	**	0.77	n.s.	15.52	**	SLX
	**Micro-**	1,154	14.43	**	265.63	***	281.91	***	SDEM

^1^ SEM: Spatial Error Model; SLX: Spatially Lagged X Model; OLS: Ordinary Least Squares regression; SDEM: Spatial Durbin Error Model; LHR: Likelihood Ratio.

^2^ p-val (p-values)

***: <10^−3^

**: <10^−2^

*: <5.10^−2^;.: <10^−1^; n.s.: >10^−1^.

^3^ PAN: Pantropical; ZAM: Zambia; ECU: Ecuador; PHI: Philippines.

We could not ratify the need of a spatial model in the samples at the macro-level for individual countries, but this was confirmed for the other nine samples (5%-significance threshold). In five of them, the existence of neighbor interactions (spatially lagged X) was demonstrated and either SLX or SDEM was selected as the best model. This was the case for all the micro- and meso-level specifications for Ecuador and the Philippines. Furthermore, in eight of the twelve samples, a model which accounts for spatially dependent errors was selected, namely SEM or SDEM. These eight cases include all the specifications for the aggregated pantropical models (at the three spatial levels), and all the specifications at meso- and micro-level for individual countries excluding the Philippines’ meso-level. In summary, from the twelve samples: three were not specified to any spatial model (and thus remained as OLS), one was assigned to a SLX model, three were acknowledged as a SEM model, and five were specified as a more complex SDEM model (all the micro-level samples and Ecuador’s meso-level sample).

### 3.4 Model performance

Table A in S3 File provides a detailed list of global measures for the OLS and the spatial models. The OLS models have an adjusted R^2^ between 0.70 and 0.95 and high statistical significances according to the results of the F-test. Just one sample, Zambia’s macro-level, with only nine sample units, presented lower (but still significant) F statistics. In the case of the nine selected spatial models, the adjusted coefficients of determination range between 0.74 and 0.94, the highest being Philippines’ micro-level and the lowest being the meso-level of Zambia. Only samples at Zambian meso- and micro- levels have a lower explanatory power (adjusted R^2^) compared to the respective tests including data from all countries. The number of degrees of freedom of the spatial models was reduced with respect to the OLS models, in a number equal to the newly introduced parameters (one degree for the lambda error—in SEMs and SDEMs- and one degree for each variable of the model—in the SDEMs and SLXs-). The ranges for standard regression errors (SER) in the spatial models vary from 0.37 in Philippines meso-level until a maximum of 0.66 in Ecuador’s micro-level. The values for the inversed logarithmic likelihoods (logLik), for the Akaike and Bayesian information criterions (AIC, BIC), for the unbiased estimator of the error variance (ML_unb_) and for the SERs increased gradually at smaller spatial levels within each countries’ samples. By this, we can see that the models at smaller spatial levels perform better. The only exception to this was the ML_unb_ and SER values for Zambia’s spatial model at micro-level, which decreased when compared to the meso-level.

**Fig 4:**
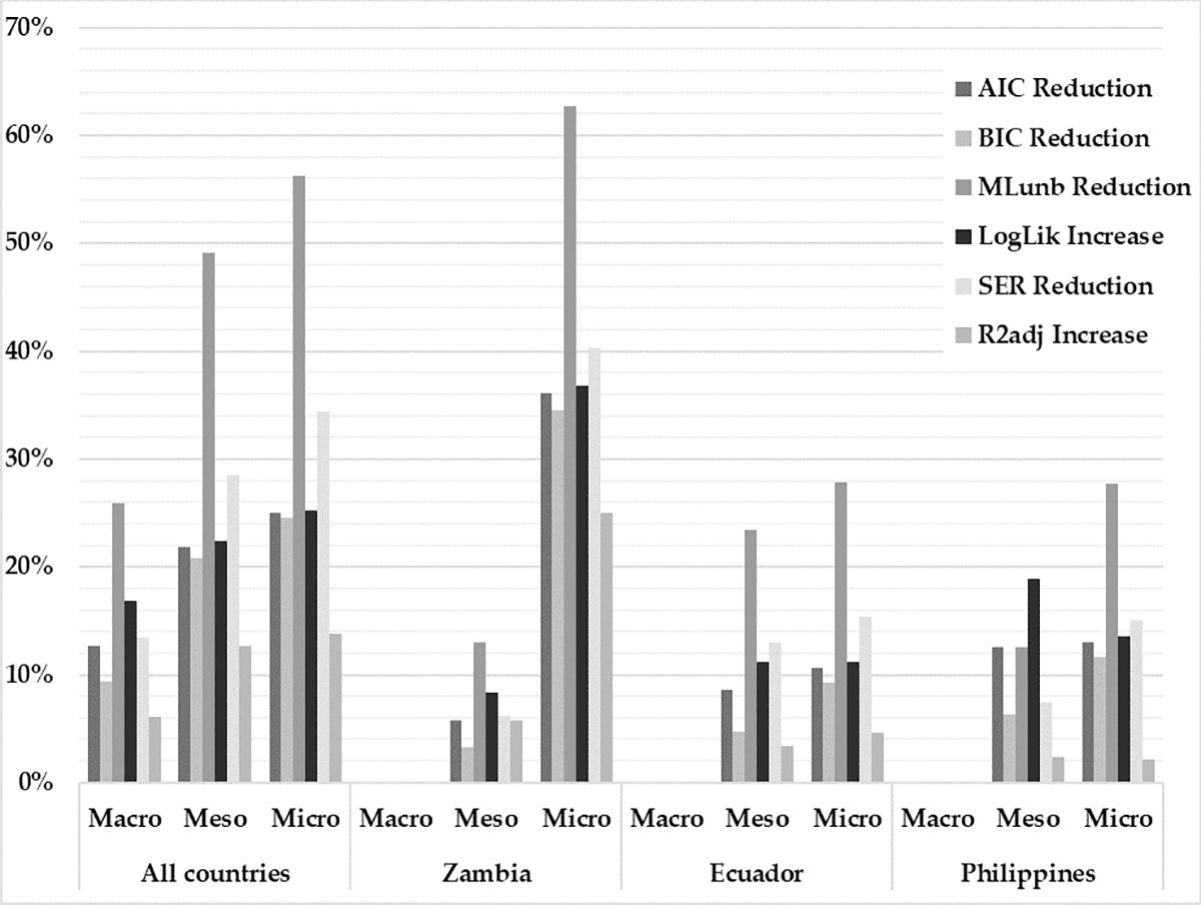
Improvement of the global measures for the spatial models compared to the respective OLS models: relative (in %) increase or reduction.

Fig 4 shows how all the global measures were improved by the nine selected spatial models. This is displayed as relative increase or decrease of the original OLS parameter values. It is clearly noticeable that most of these proportional improvements gradually grow at the lower levels. This growth is especially strong in the micro-level SDEM in Zambia, where the highest values are observed for all the measures, while the SEM for the meso-level presented some of the lowest relative improvements. The models with the smallest improvements were the ones at the meso-level for individual countries (SEM in Zambia, SDEM in Ecuador, and SLX in Philippines) and the country-specific models at the macro-level by omission of spatial specification.

### 3.5 Spatial regression models

Tables 6 and 7 show the regression results of the specified models for the pantropical samples cross-scale and for the micro-level samples cross-country, respectively. In order to interpret the influence of the regression determinants on FC, all coefficient signs have to be reversed due to the transformation of the dependent variable (FC*).

**Table 6:** Impacts for aggregated pantropical samples in specified spatial models.

		Macro-level (SEM)				Meso-level (SEM)				Micro-level (SDEM)			
		N = 49				N = 361				N = 3,035			
		Coef	SE	z- Val	P> |z|	Coef	SE	z- Val	P> |z|	Coef (Total)	SE	z- Val	P> |z|
**Pantropical models**	***λ*(Err)**	0.51	0.13	3.97	***	0.72	0.04	18.99	***	0.72	0.01	56.18	***
	**Inter**	0.70	0.13	5.59	***	1.08	0.10	10.46	***	1.57	0.04	41.65	***
	**A**_**TOT**_^**^**^	x	x	x	x	-0.09	0.05	-1.87	*	0.08	0.04	2.07	*
	**PVA**^**^**^	-	-	-	-	0.19	0.04	4.76	***	0.03	0.03	0.94	n.s.
	**PP**_**FA**_^**^**^	1.19	0.09	14.01	***	1.63	0.05	31.90	***	1.89	0.04	45.96	***
	**RD**^**^**^	-	-	-	-	-	-	-	-	-	-	-	-
	**FL**^**^**^	x	x	x	x	x	x	x	x	x	x	x	x
	**CSI**^**^**^	0.27	0.07	3.97	***	0.21	0.04	5.37	***	0.28	0.36	7.87	***
	**CY**^**^**^	x	x	x	x	xx	xx	xx	xx	xx	xx	xx	xx

Coef: Coefficient; SE: Standard Error; x: variable eliminated by de model; xx: not applicable in this model; -: Collinearity > 0.6

^: linearized and standardized–variable

***: <10^−4^

**: <10^−2^

*: <10–1; n.s.: >10^−1^.

**Table 7:** Impacts for country-specific (and aggregated) samples at micro-level.

		SDEM—Spatial Durbin Error Model											
		Direct impacts: observed unit				Indirect impacts: neighboring units (lag X)				Total impacts			
		Coef	SE	z-Val	P>|z|	Coef	SE	z-Val	P>|z|	Coef	SE	z-Val	P>|z|
**Zambia** **N = 1,016**	**Inter**	0.87	0.06	13.70	***				***λ*(Err)**	0.79	0.02	40.04	***
	**A**_**TOT**_^**^**^	x	x	x	x	x	x	x	x	x	x	x	x
	**PVA**^**^**^	-0.09	0.02	-4.45	***	-0.04	0.05	-0.77	n.s.	-0.13	0.05	-2.45	*
	**PP**_**FA**_^**^**^	1.21	0.03	35.16	***	-0.09	0.06	-1.63	n.s.	1.11	0.06	18.45	***
	**RD**^**^**^	0.01	0.02	0.47	n.s.	-0.12	0.05	-2.55	*	-0.11	0.06	-1.91	*
	**FL**^**^**^	x	x	x	x	x	x	x	x	x	x	x	x
	**CSI**^**^**^	0.15	0.02	7.09	***	0.22	0.05	4.48	***	0.37	0.06	6.59	***
	**CY**^**^**^	xx	xx	xx	xx	xx	xx	xx	xx	xx	xx	xx	xx
**Ecuador** **N = 865**	**Inter**	1.26	0.05	25.62	***				***λ*(Err)**	0.54	0.04	15.58	***
	**A**_**TOT**_^**^**^	x	x	x	x	x	x	x	x	x	x	x	x
	**PVA**^**^**^	0.21	0.03	6.58	***	-0.14	0.06	-2.49	*	0.07	0.06	1.15	n.s.
	**PP**_**FA**_^**^**^	1.86	0.04	49.84	***	0.25	0.06	4.27	***	2.12	0.06	37.33	***
	**RD**^**^**^	-	-	-	-	-	-	-	-	-	-	-	-
	**FL**^**^**^	-0.09	0.05	-1.61	n.s.	-0.15	0.07	-2.04	*	-0.23	0.05	-4.29	***
	**CSI**^**^**^	0.09	0.03	2.72	**	0.28	0.06	4.62	***	0.36	0.06	5.86	***
	**CY**^**^**^	xx	xx	xx	xx	xx	xx	xx	xx	xx	xx	xx	xx
**Philippines** **N = 1,154**	**Inter**	2.44	0.03	81.80	***				***λ*(Err)**	0.50	0.03	18.42	***
	**A**_**TOT**_^**^**^	0.21	0.02	9.37	***	0.11	0.04	3.23	**	0.32	0.04	8.03	***
	**PVA**^**^**^	0.40	0.02	19.34	***	-0.04	0.02	-1.91	*	0.35	0.03	12.05	***
	**PP**_**FA**_^**^**^	2.32	0.02	96.96	***	0.04	0.04	1.07	n.s.	2.36	0.04	54.17	***
	**RD**^**^**^	-0.31	0.02	-13.18	***	0.07	0.04	1.75	*	-0.24	0.04	-5.71	***
	**FL**^**^**^	x	x	x	x	x	x	x	x	x	x	x	x
	**CSI**^**^**^	x	x	x	x	x	x	x	x	x	x	x	x
	**CY**^**^**^	xx	xx	xx	xx	xx	xx	xx	xx	xx	xx	xx	xx
**Pantropical** **N = 3,035**	**Inter**	1.57	0.04	41.65	***				***λ*(Err)**	0.72	0.01	56.18	***
	**A**_**TOT**_^**^**^	0.01	0.02	0.79	n.s.	0.07	0.03	2.10	*	0.08	0.04	2.07	*
	**PVA**^**^**^	0.18	0.02	11.07	***	-0.15	0.02	-6.16	***	0.03	0.03	0.94	n.s.
	**PP**_**FA**_^**^**^	2.14	0.02	101.87	***	-0.26	0.04	-7.13	***	1.89	0.04	45.96	***
	**RD**^**^**^	-	-	-	-	-	-	-	-	-	-	-	-
	**FL**^**^**^	x	x	x	x	x	x	x	x	x	x	x	x
	**CSI**^**^**^	0.12	0.02	7.34	***	0.17	0.03	5.66	***	0.28	0.36	7.87	***
	**CY**^**^**^	xx	xx	xx	xx	xx	xx	xx	xx	xx	xx	xx	xx

Coef: Coefficient; SE: Standard Error; x: variable eliminated by de model; xx: not applicable in this model; -: Collinearity > 0.6

^: linearized and standardized–variable

***: <10^−4^

**: <10^−2^

*: <10–1; n.s.: >10^−1^.

**Fig 5:**
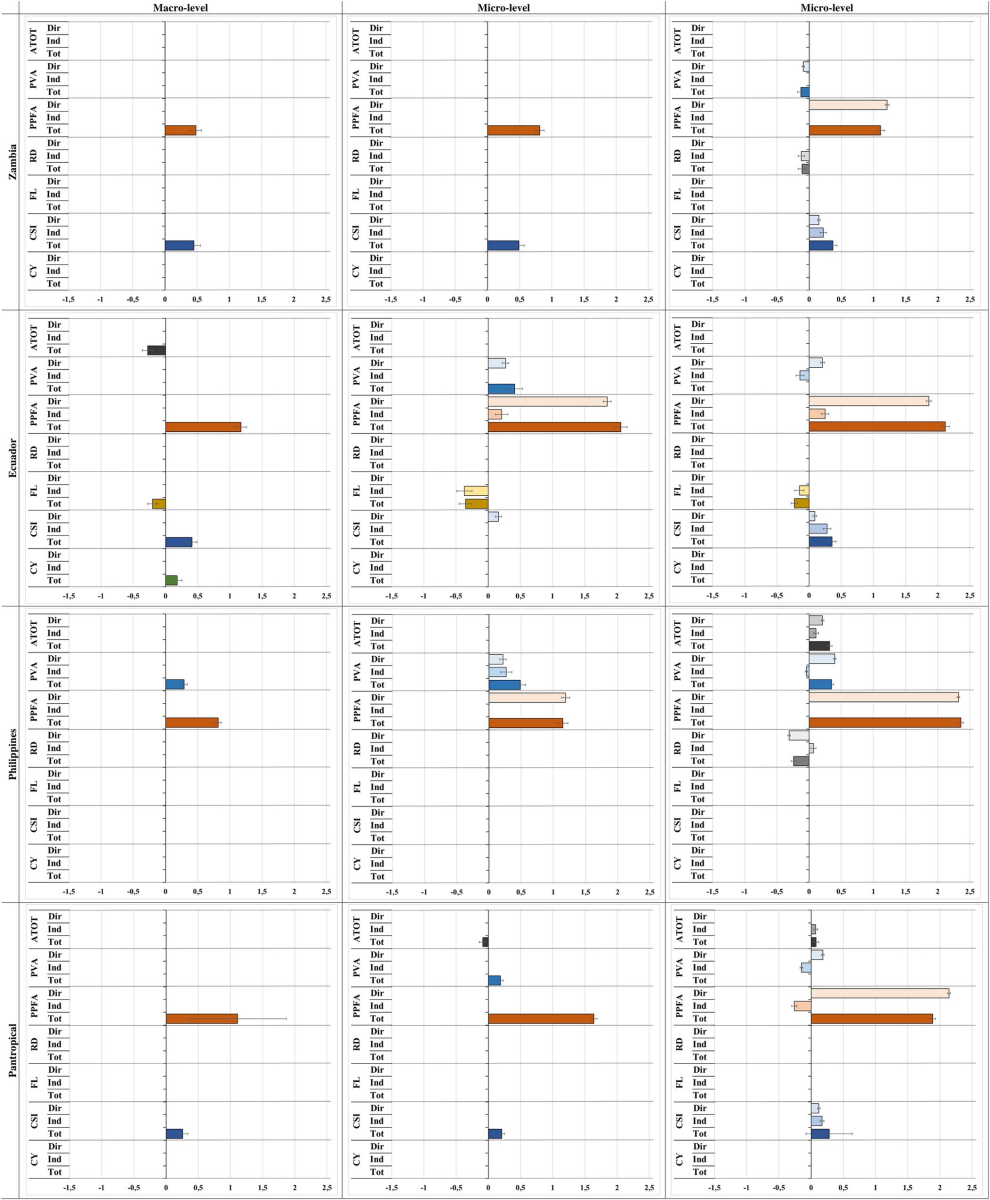
Coefficients and standard errors of the seven explanatory variables (drivers of deforestation) of the selected models for the twelve samples, across spatial level and country context.

Fig 5 provides a visual summary of the coefficients (and standard errors) of the seven variables in the selected models for the twelve samples (across level and country). Only the results of the determinants, which showed a significant contribution (considering a p-value threshold of 10%), are shown.

Only significant parameters (p-value threshold of 10%) are shown. Variables are linearized and standardized. In the case of non-spatial and spatial error models, the coefficients or effects are displayed as total impacts. (Dir: Direct impacts. Ind: Indirect impacts (neighbors). Tot: Total impacts.) (ATOT: Total Area [ha]. PVA: Share of potential vegetation on surface area [%]. PPFA: Population pressure on remaining forest area [pers./ha]. RD: Road density [km/km2]. FL: Flatness: share of surface with less than 16% steepness [%]. CSI: Crop suitability index [%]. CY: Maximum cereal area yield [kcal/ha].)

Additionally, we provide Tables B-G in S3 File to assist during the analysis of this study’s results. Namely, we compiled a detailed and comprehensive table on the descriptive statistics for all the variables used in each of the samples (Table B in S3 File), the results of the collinearity check (Table C in S3 File), the results for the simple regressions (Table D in S3 File), and the results for all OLS models and the non-significant spatial models for macro-, meso- and micro-level (Tables E, F and G in S3 File, respectively).

We can observe that the number of significant explanatory variables, the values of error term parameters (*λ*), and neighboring effects (*θ*), tend to increase at smaller administrative levels, in both the aggregated (Table 6) and country-specific models (Tables F and G in S3 File and Table 7).

Across all spatial sales and countries, PP_FA_ always depicts the strongest contribution to all models with the highest coefficients (around three to ten times larger than the other variables) and a negative influence on FC. The relative contribution of PP_FA_ to the models is gradually increasing at smaller levels, both in the pantropical models (from 1.19 to 2.14) and in the country-specific models. Together with PP_FA_, either CSI or PVA are also present in the models for all the samples. On the one hand, CSI is significant in all pantropical samples, but with slightly smaller coefficients at lower spatial levels. This determinant always has a negative contribution to FC in all the models were it is significant (nine out of the twelve). On the other hand, PVA was significant in eight of the twelve samples (mostly meso- and micro-levels) with negative influence on FC in seven of those. The other variables showed a more differentiated pattern across scale and contexts. A_TOT_ presented smaller but significant impacts on FC with varying signs in only three of the twelve models. FL was included in all Ecuador-specific models only, where it influences FC positively. From the five samples where CY was available, it only had a significant (negative) effect on FC in the macro-level OLS model for Ecuador. We detected a strong collinearity (correlation above 0.6, see Table C in S3 File) between RD and PP_FA_ in ten of the twelve samples. Thus, this variable was only included (and found significant) in two country-specific samples at the micro-level. The samples for Zambia’s macro and meso-levels further showed strong collinearity between other variables, for instance between A_TOT_ and CSI, PP_FA_ or RD and between RD and CSI.

When observing the results for the micro-level analysis, where a SDEM including spatial errors and indirect impacts from neighbors was always specified (Table 7), we can identify more context dependencies. Neighbor effects are also observed in Philippines’ and Ecuador’s meso-level results. Although in general the total effects of the spatial models are similar, in direction and intensity, to those of the OLS models, we can see some particular exceptions. The variable RD in Zambia’s micro-level, for example, loses its high significance in the SDEM model. In other cases, the indirect effects of the neighbors represent a relatively large or even more significant contribution to a variables’ behavior than the direct effects. We can see this in Ecuador’s results, for CSI and FL, and for Zambia’s CSI as well as for the pantropical results’ CSI and A_TOT_. This is also true for Ecuador’s FL and for Philippines PVA at meso-level. In some other cases, even the direction of the indirect impacts differs from the direct effects, while still in notable intensities and significance. This happens for PVA in Ecuador’s and in the aggregated results, PP_FA_ in the aggregated model, and RD in the Philippines sample; all of them, at the micro-level. At the micro-level, the strongest and most significant effect of neighboring regions is observed in Ecuador and in the pantropical model.

In general, the error coefficients were higher in the pantropical models (0.51 to 0.72) compared to the respective country-specific models (0.43 to 0.54). The only exception to this is the highest spatial error coefficient (*λ*), which was identified in Zambia’s micro-level sample (0.79). Moreover, the spatial error terms are relatively large in all SEM and SDEM models if compared to the other variable coefficients (except PP_FA_).

## 4 Discussion

### 4.1 Insights from spatial econometrics

#### 4.1.1 Econometric models of pantropical deforestation and spatial dependencies

We calculated highly significant econometric models with cross-section data applying a sigmoid function for different spatial levels and tropical contexts. This represents a first empirical attempt in scientific research on basis of an aggregated pantropical study. Both non-spatial and spatial regressions resulted in significant models of deforestation for different countries and jurisdictional levels (see Table A in S3 File).

However, we demonstrated spatial dependency and consequently, the use of spatial models was justified in at least nine of the twelve studied samples (Tables 4 and 5). Our results support Tobler’s first law of geography [114], which implies that social and physical events are highly clustered in space. In these nine cases (all except for the country-specific macro-levels), the inclusion of spatial errors and/or spatially lagged Xs improved the explanatory power and the goodness of fit of the spatial models significantly. The application of this theory to drivers of deforestation has not yet been addressed intensively in empirical research. Our results indicate that neglecting spatial effects in this kind of studies can lead to several problems and misinterpretation, e.g. bias of coefficients, falsely classified predictors, or even opposite change of effect directions.

Before discussing our general findings into detail, we will highlight some of the methodological limitations and the potential for innovation of this study.

#### 4.1.2 Methodological limitations and innovation

Understanding the influence of the spatial scale on drivers of deforestation constitutes a methodological and conceptual challenge. Especially, if generalizations on FC and deforestation rates are to be used as a framework for operationalizing adequate forest policies like REDD+ [115,116]. First, the assumptions and restrictions of complex spatial econometric models require cautiousness when interpreting causality or inference. For example, the chosen variables related to the most common drivers of deforestation can have some degree of endogeneity ([13,117]). Increasing population needs more roads, agricultural land and thus, results in reduced FC. But, at the same time, in areas with middle to high FC, as deforestation increases, more roads can be built, more land can be cleared and, thus, the number of people that can be supplied increases as well. Moreover, we detected and treated persistent collinearity (especially strong in Zambian samples), mostly between population and other variables, like road density. This warns us to be especially careful with the analysis of these cases and suggests the thorough exploration of the relations between selected determinants in similar approaches. Besides, due to the nested multi-level design of our study, our samples had very different sizes ranging from 9 to over 3,000 units, which has to be kept in mind again when comparing the respective results.

As often discussed, the results of spatial models can be very different depending on the neighbor matrix and the specified model [118]. The spatial weights matrices (*W*s) based on the SOI method resulted to be an improved alternative to reflect the spatial interactions in the twelve very heterogeneous samples [118,119]. As with other graph-based matrices such as Delaunay triangulation or Gabriel *W*, SOI presented advantages to other–and more commonly used–contiguity (e.g. rook, queen) or distance-based *W*s. For instance, all the resulting *W*s were symmetric (if *i* is neighbor of *j*, *j* is neighbor of *i*), and row standardization allowed for proportional weights when features had unequal number of neighbors. Moreover, this type of spatial weight does not need a common border between units, which allowed us to work with close island regions as neighbors like in the case of the Philippines. Moreover, this type of matrix enabled working with separated blocks, for instance treating and comparing different countries in the aggregated sample. The SOI matrix and a local model specification fulfilled the purposes of our study providing relevant answers to our research questions.

Our proposed econometric models deal with the issue of spatial autocorrelation or spatial dependency of the observations (endogenous/exogenous variables or error terms). Spatial autocorrelation exists due to a diversity of phenomena related to measurement (choice of observation unit), externalities or spillovers. Our selected nested models allowed us to compare impacts between parameters and provide generalized spatial models of deforestation, which could be compared to each other as well. However, further studies with similar approaches could explore other types of commonly used spatial regression models, which present other advantages or possibilities for the analysis. For example, gobal spatial models (such as the classical Spatial Durbin Model) are based on simulations and imply more complex interactions, as the changes in neighbors’ FC affect the FC in all the system units [120,121]. Another option are geographically weighted regression (GWR) models, which deal with another kind of spatial phenomena–although related to autocorrelation-, namely: spatial heterogeneity, heteroscedasticity, spatial non-stationary or structural instability in space. Therefore, in GWR models, the explanatory variables can have different effects or parameters at different points, and the error term may vary spatially as well. This is why GWR models are normally considered as a good exploratory method to visualize non-stationary phenomena [122–124].

Other options for further analysis could be the use of spatial panel data models or laying the focus on deforestation rates as dependent variable [120,125]. An analysis of this type could reflect the forest trajectory of each particular administrative unit, but it would imply further methodological compromises. For instance, it would need harmonized information about land (forest) cover and its drivers for the three countries at different points in time. Including more variables or indicators for drivers of deforestation could also brighten the opportunities for further analyses. On the one hand, the availability and the quality of the existing information vary significantly across spatial and jurisdictional levels. Some of this information is normally missing or problematic to obtain at subnational levels, especially in developing tropical countries. For instance: economic, agricultural or land use data are normally collected and summarized at national or regional level for international reporting, or sometimes available but in different formats or quality standards (e.g. CSI and CY). Another example is the reliability of the available administrative boundaries themselves in resembling the actual political scenarios on the ground. Normally, official governmental boundaries do not acknowledge customary management and governance schemes, such as chiefdoms in Zambia, indigenous territories in Ecuador or ancestral domains in the Philippines [126,127]. Moreover, this boundaries are frequently unsure or inaccurate, due to a combination of an on-going modernization of the technical mapping capacities of the relevant institutions, and in some cases, regional tensions and disputed boundaries [128,129]. But on the other hand, the existing data on global deforestation and related drivers (e.g. [1,72,130]) is constantly increasing and more new sources are regularly updated at medium to high resolution. This fact creates promising room for innovative approaches and new research directions.

### 4.2 Scale and context dependency of deforestation drivers

#### 4.2.1 Constant leading impact of population pressure and land suitability for agriculture

As expected and in line with previous studies [13,26], we empirically identified population pressure (i.e. PP_FA_) and land suitability for agriculture (CSI or PVA) as the main recurrent pantropical drivers of deforestation. While previous studies have been focusing on specific administrative levels or specific countries, our models show that this phenomenon occurs across all jurisdictional levels and different tropical contexts.

Moreover, demographic pressure, which expresses the need for agricultural land and infrastructure at the expense of natural resources, has by far the highest (negative) influence on FC. Its standardized impacts are five to ten times larger than those for the other significant determinants. Thus, our study not only confirms the constant negative impact of population pressure on FC, but it empirically demonstrates its leading influence across different regions and jurisdictional levels. These results suggest, that it is the best ‘stand-alone’ indicator for FC change across contexts and scales [30,131,132].

The influence of demographics is combined and intensified by the natural conditions of the land, expressed by the crop suitability index and the share of potential vegetation area. Constantly influential across scale and context, higher land suitability for agricultural production triggers the conversion of forests to such. This highlights the importance of competition for land between different forest and alternative land uses–and their respective opportunity costs–as a recurrent universal phenomenon in forest-agriculture frontiers [133,134].

#### 4.2.2 Scale dependency: Heterogeneity at local levels

We could recognize recurrent and clear differences across spatial levels. In general, the number of explanatory variables increases at more local scopes, in both the aggregated and country-specific results. Moreover, the quality and the statistical significance of the models increases with smaller administrative units, while their explanatory power decreases. This is due to the heterogeneity and the larger sample size of the lower levels, which is not yet completely explained by the tested variables. In addition, strong spatial errors and larger Moran’s *I*s of the residues, suggest that important spatially correlated variables might have been omitted, especially at the lower levels. Smaller jurisdictional units require more complex models, which account for both larger spatial errors and stronger neighbor interactions [37]. Our results confirm the evidence from the literature and previous studies, which is rich in local-scale cases that exhibit complex patterns and processes of coupled human and natural systems [10,13,135].

Furthermore, the studied drivers influence FC with varying intensities and directions depending on scale and the regional context. The impact of demographics, for instance, gains strength at smaller administrative units across contexts, while the variables associated with agricultural suitability (CSI for Ecuador, Zambia and pantropical models, PVA for Philippines’ model), present similar ranges of intensities across scales. This could be representing a more direct pressure on the natural resources and signifying the human competition for land in systems with narrower limits [136]. Likewise, when analyzing the results of the pantropical models, the area size of the administrative unit apparently has a negative influence on forest at the micro-level, but positive at the meso-level. Although this impact is relatively little if compared with the ones of other determinants, it is related to the existence of small units within urban areas at the micro-level. According to our definitions, these areas result in high FC as most of its limited potential vegetation area comprises natural parks and tree areas, but little pastures, crops or grasslands.

#### 4.2.3 Context dependency: Particularities of the countries

Some context-specific findings of our study can be highlighted. The larger error coefficients (*λ*) of the pantropical models indicate the importance of some omitted variables that account for these contextual differences. The missing factors could be related to regional, geographic or ecological dissimilarities of the three countries.

In the case of Zambia, the variable potential vegetation area had a positive influence on FC. This might be representing the relevance of woodland areas and shrubs and their compensation effect when being used or classified as forests [126,137]. Another possible explanation could be the land cover information used, which is generalized for Africa and does not distinguish between the varying and complex forest ecosystems in the country, ranging from evergreen closed forests to open miombo or mopane woodlands and bushlands [58,138,139]. Furthermore, the lower quality and explanatory power of the models, together with their higher spatial errors (especially at the micro-level), clearly suggest that the models could not capture another determinant, which is less relevant at district or province level. As suggested by other authors [46,58], this could be related to the existence of more local events such as fire occurrence or wood extraction for charcoal and fuel production. Furthermore, we observe a less important role of demographics in comparison to Ecuador and Philippines, most likely due to the lower population density.

Besides, Ecuador has the models with the most significant independent variables, explaining the heterogeneity of the country at all spatial levels from an ecological and socio-economic perspective. Flatness, for example, was positively correlated with deforestation at all spatial levels, only in this country. This might be due to the more diverse geographic conditions and the larger differences between the steep Andean slopes with historical deforestation and the lowland areas as a current deforestation frontier [140,141]. Similarly, cereal yield was significant at the macro-level of Ecuador only, and associated with deforested provinces. At this large-scale picture of the country, cereal yields for maize and rice are much higher in the coastal and central areas, where the cultivation of these crops is more extended and more commercially oriented [142]. In these provinces, relatively little or almost none forest is left. At the same time, indigenous groups with a more subsistence oriented crop production inhabit large areas of the Amazon with lower agricultural yields [142,143]. These results might reflect the importance of effective and conscious territorial organization [144], like the particular governance schemes taking place at the different spatial levels in the country [127,145].

The models for the Philippines included the smallest number of significant explanatory variables, and population pressure is apparently explaining FC change almost exclusively. It is important to understand the archipelago condition of this highly populated country, plus its late/post- transition context, in which massive deforestation has already taken place resulting in the actual national forest-agriculture mosaic [146,147]. This might also be the reason why factors like the crop suitability index are not significant, in contrast to the other countries where higher deforestation rates are still observed. However, the uniform and substantial contribution of the share of potential vegetation area to the model might be capturing this influence of key deforestation and degradation drivers, such as agricultural expansion or forest product extraction [148]. A last remark must be made regarding the omission of flatness in the three spatial levels. This is observed despite the biophysical heterogeneity of the Philippines, a country which has even been using a slope threshold to legally define forestland [149]. For decades, all areas above 18% of slope have been classified by the Philippine institutions as forestland regardless of whether any tree cover was present, because of their location in mountain ranges or in hardly accessible areas where forest was (usually) found [150].

#### 4.2.4 The impacts of neighbors: Defining the system limits

It is important to understand that the effects of many socioeconomic, political and ecological drivers of de- and reforestation are often perceived at other spatial levels or at different jurisdictional units than those ones where the actual causes are being generated [17]. For example, national, regional or global decisions from private and/or public actors regarding forests and agriculture (e.g. trade agreements or conservation policies), might help intentionally or not in halting or increasing deforestation at different smaller geographical contexts [151–153]. Similarly, but on the opposite direction, both community decisions referring to priority areas for protecting forest functions, as well as land use/cover changes related to local income or opportunity costs, might turn relevant on provincial, national or international levels, sometimes even in conflict with private or governmental interests [2,154,155]. These connections between neighbors and hierarchies are not always easy to identify, quantify and weight, as they are a miscellaneous result of geographical, historical, political, economic and even random conditions that may vary from region to region.

The results for the indirect impacts provided by the spatial models at the smaller levels offer some interesting insights and room for discussion. For instance, the neighbors’ suitability for crop production (in Ecuador and Zambia) even has a stronger influence on FC than the unit of analysis itself. We can observe the exact same behavior between adjacent units (stronger impacts of neighbors) in Ecuador with other variables of higher resolution, like flatness or population. Moreover, we also identified these interactions in the results for the potential vegetation area in the Philippines’ at meso-level. In some other cases, the neighbors influence FC with inverse directions. If we analyze the pantropical model, for example, larger potential vegetation areas and population densities in the neighboring units apparently release the pressure on forest. Furthermore, the influence of neighboring units on deforestation at meso-level appears to be more significant in Ecuador and Philippines than in Zambia. Perhaps, because the smaller size of the counties (Ecuador) and provinces (Philippines) allows these interactions to happen, if compared with the larger districts in Zambia. Other reasons could be the contrasting geography of the countries, the obvious differences on connectivity (e.g. islands vs. landlocked) and infrastructure, or other data-driven explanations such as the use of a non-realistic neighboring matrix and the quality of Zambia’s unofficial boundary dataset [52]. Moreover, these neighbor interactions between provinces and administrative regions do not seem to be relevant at the macro-level in any of the countries, individually or aggregated.

In any case, our results are empirical evidence that certain deforestation forces occur independently of de jure governance boundaries; thus, they should be addressed setting broader and more flexible system limits, which consider the complex socio-ecological characteristics of each particular landscape.

### 4.3 Policy implications: The scale of REDD+

Policy design usually takes place on different interacting levels, such as international conventions, national laws, regional policy programs and local on the ground initiatives. The degree of federalism and decentralization differs among countries. Developing countries often have highly complex laws and regulations, which are, however, frequently inconsistent among concurring policy resorts or governance hierarchies and therefore unclear. Additionally, low capacities and weak law enforcement impede the governance process [156,157]. A clear example of this are the challenges and difficulties often faced during the design and implementation of REDD+ programs on the ground [158–160]. In order to ensure the success of such measures and reach both global and local objectives, there is a need for coordinated coherent policy design [14,160,161].

Our results indicate that anticipating demographic development and harmonizing forest and agricultural policies with increasing population pressure are of highest priority at all spatial levels and across countries. However, the strikingly strong relationship between demography and FC could indicate clear limitations of sectoral policy far beyond forestry, agriculture or even beyond bioeconomy. Although the main drivers of tropical deforestation are strongly dominated by socio-economic factors (e.g. demographic and infrastructure development), they are sensitive to the context and spatial scale, thus being case specific. Our findings stress the importance of taking context-specific factors into account, especially at smaller spatial scales. The varying spatial interactions between neighbors and drivers suggest a demand for flexibility when setting system boundaries in forest-related policy. Thus, depending on the specific tropical context and scale, a different spatial focus (beyond the existing *de jure* governance configurations) might be needed, in order to design effective measures, which halt deforestation.

Our results highlight the need for coherence between forest conservation and management policy implementation from national to local level on the one side. On the other side, they signalize the need for suitable demographic and agricultural policies across scales and countries. These raises some questions in line with frequent discussions [16,160], such as how sustainable and efficient conservation and restoration measures can be in highly populated areas or in societies with weak governance.

## 5 Conclusions

Our study represents a first attempt of generating econometric models of pantropical deforestation that consider subnational administrative units. We were successful in providing highly significant models that quantify the influence of commonly identified drivers of deforestation for different tropical contexts and spatial levels. We also demonstrated that neglecting spatial effects in this type of studies can lead to several problems or misinterpretations.

We conclude from our findings that the enforcement of policy instruments should start from common entry points at the international level and has to be then modified and adapted to particular national, regional or local conditions. International and national policy makers should focus on addressing demographic/infrastructure development and overcoming conflicts with agricultural purposes, while designing the framing conditions for efficient land use planning and policies. This can only be effective if global, national (large scale) REDD+ policy leaves enough flexibility for smaller scale adaptation of the policy frameworks to the respective socio-ecological conditions. Some successful examples of this are decentralization efforts such as ‘landscape approaches’ or participatory and community-based forest management, as long as broader national and international political commitment is present [162,163].

## Supporting information

S1 Fig Forest transition phases according to different categorizations and expected situation of the selected countries within the forest transition curve. Forest Cover (FC) vs. Socio-economic development (above). Annual Forest Change Rate (AFCR) vs. Socio-economic development (behind): (a) FAO (2015)–FC: Forest cover; AFCR: Annual forest change rate. (b) Angelsen and Rudel (2013)–Quote: “The FT framework suggests that over time a country (or region) moves through three stages: (1) high forest cover and low deforestation (“core forests”), (2) accelerated deforestation and shrinking forest cover (“frontier forests”), and (3) stabilization and eventual reversal of the deforestation process (“forest-agricultural mosaics”)”. (c) da Fonseca et al. (2007)–HFLD: High FC (>50%), Low Deforestation rate (AFCR > -0.22%/yr.)–HFHD: High FC (>50%), High Deforestation rate (AFCR < -0.22%/yr.)–LFHD: Low FC (<50%), High Deforestation rate (AFCR < -0.22%/yr.)–LFHD: Low FC (<50%), Low Deforestation rate (AFCR > -0.22%/yr.). (d) Hosonuma et al. (2012) Pre-transition: FC>50% and AFCR > -0,25%, Late transition: FC < 15% or AFCR = 0% or decreasing AFCR, Post-transition: FC < 50%, Early transition: Remaining cases.
(TIF)

S1 File Geodatabase. Compressed file including Excel tables and ESRI shapefiles with the variables for all the samples.
(7Z) Click here for additional data file.

S2 File R Script used for statistical analysis. (R)

S3 File Supporting information. Table A: Global measures of the models. Table B: Descriptive statistics. Table C: Multi-collinearity results. Table D: Simple linear regressions. Tables E, F, G: Impacts for the additional OLS and spatial models.
(PDF)

S4 File Executive summary of the main findings. (PDF)
