# Supplementary figures and images for: Scale and context dependency of deforestation drivers: Insights from spatial econometrics in the tropics
Source: PLoS One. 2020 Jan 29;15(1):e0226830. doi: 10.1371/journal.pone.0226830 (PMC6988916; doi:10.1371/journal.pone.0226830)

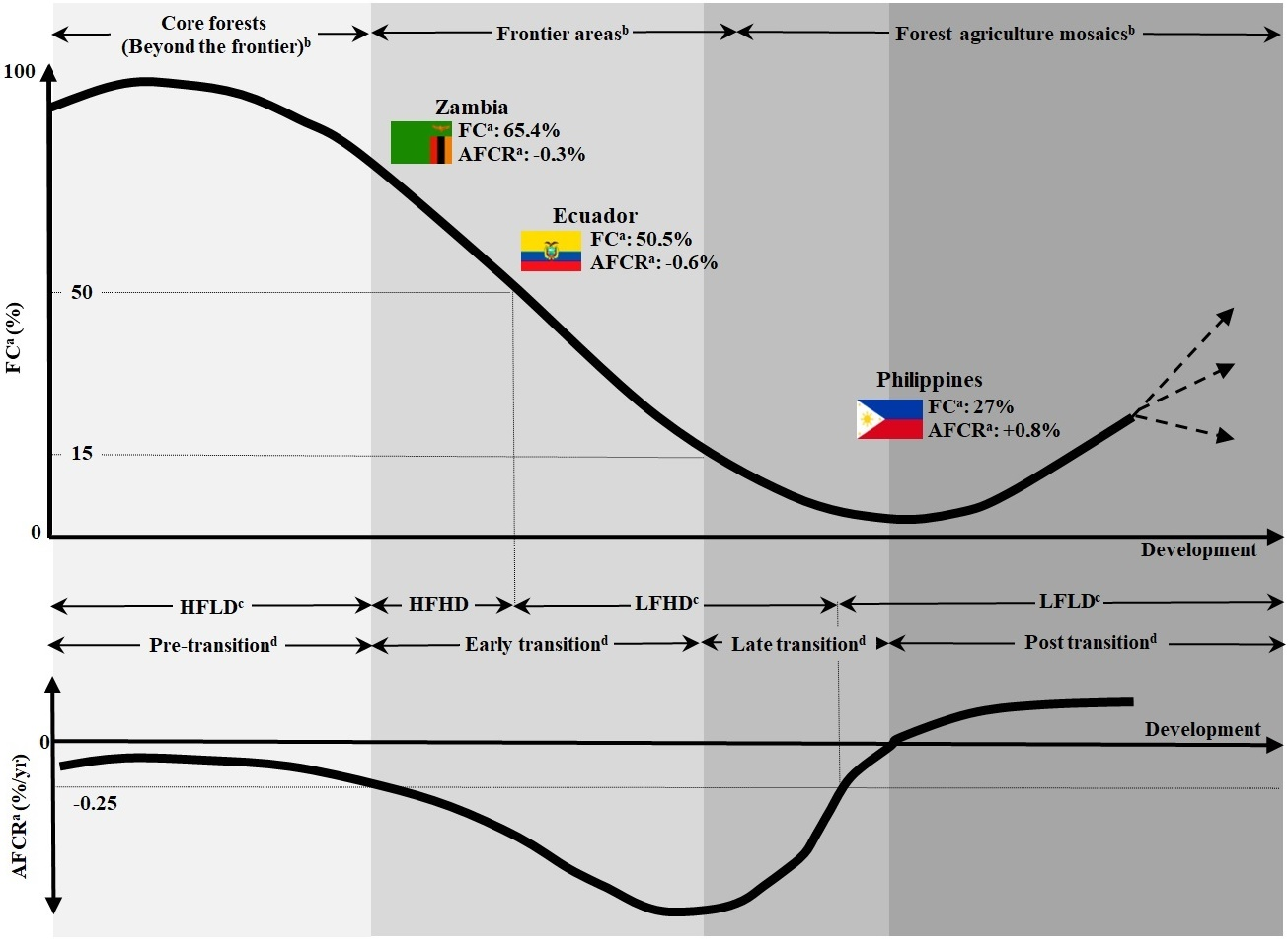

Supplement: S1 Fig — Forest Cover (FC) vs. Socio-economic development (above). Annual Forest Change Rate (AFCR) vs. Socio-economic development (behind): (a) FAO (2015)–FC: Forest cover; AFCR: Annual forest change rate. (b) Angelsen and Rudel (2013)–Quote: “The FT framework suggests that over time a country (or region) moves through three stages: (1) high forest cover and low deforestation (“core forests”), (2) accelerated deforestation and shrinking forest cover (“frontier forests”), and (3) stabilization and eventual reversal of the deforestation process (“forest-agricultural mosaics”)”. (c) da Fonseca et al. (2007)–HFLD: High FC (>50%), Low Deforestation rate (AFCR > -0.22%/yr.)–HFHD: High FC (>50%), High Deforestation rate (AFCR < -0.22%/yr.)–LFHD: Low FC (<50%), High Deforestation rate (AFCR < -0.22%/yr.)–LFHD: Low FC (<50%), Low Deforestation rate (AFCR > -0.22%/yr.). (d) Hosonuma et al. (2012) Pre-transition: FC>50% and AFCR > -0,25%, Late transition: FC < 15% or AFCR = 0% or decreasing AFCR, Post-transition: FC < 50%, Early transition: Remaining cases. (TIF) [file pone.0226830.s001.tif]
