# Supplementary material for: Scale and context dependency of deforestation drivers: Insights from spatial econometrics in the tropics
Source: PLoS One. 2020 Jan 29;15(1):e0226830. doi: 10.1371/journal.pone.0226830 (PMC6988916; doi:10.1371/journal.pone.0226830)
Supplement: S3 File — Table A: Global measures of the models. Table B: Descriptive statistics. Table C: Multi-collinearity results. Table D: Simple linear regressions. Tables E, F, G: Impacts for the additional OLS and spatial models. (PDF) [file pone.0226830.s004.pdf]

Table A. Global measures for spatial and non-spatial models.

Country	Level	N	Spatial	Model	F[2]	logLik[1]	AIC[1]	BIC[1]	ML_{unb}[1]	SER[1]	$R2_{adj}$[1]	df[1]
**PAN[3]**	**Macro-**	49	No	OLS	109.59***	-36.18	80.35	87.92	0.27	0.52	0.82	46
			Yes	SEM	-	-30.10	70.19	79.65	0.20	0.45	0.87	45
	**Meso-**	361	No	OLS	332.61***	-414.85	841.70	865.03	0.59	0.77	0.79	356
			Yes	SEM	-	-321.87	657.74	684.97	0.30	0.55	0.89	355
	**Micro-**	3,035	No	OLS	3,070.70***	-3,969.35	7,950.70	7,986.81	0.80	0.90	0.80	3,030
			Yes	SDEM	-	-2,968.91	5,959.82	6,026.02	0.35	0.59	0.91	3,025
**ZAM[3]**	**Macro-**	9	No	OLS	11.79*	3.93	4.15	5.33	0.06	0.23	0.84	4
			Yes	-	-	-	-	-	-	-	-	-
	**Meso-**	70	No	OLS	80.43***	-46.28	100.56	109.56	0.23	0.48	0.70	67
			Yes	SEM	-	-42.39	94.79	106.03	0.20	0.45	0.74	66
	**Micro-**	1,016	No	OLS	652.39***	-1,098.92	2,209.84	2,239.38	0.51	0.72	0.72	1,011
			Yes	SDEM	-	-694.55	1,411.10	1,465.26	0.19	0.43	0.90	1,006
**ECU[3]**	**Macro-**	24	No	OLS	98.16***	-2.70	19.40	27.64	0.10	0.31	0.95	18
			Yes	-	-	-	-	-	-	-	-	-
	**Meso-**	212	No	OLS	378.43***	-219.15	450.29	470.43	0.47	0.69	0.88	207
			Yes	SDEM	-	-194.70	411.41	448.33	0.36	0.60	0.91	202
	**Micro-**	865	No	OLS	1,320.97***	-1,012.81	2,037.62	2,066.19	0.61	0.78	0.86	860
			Yes	SDEM		-899.71	1,821.42	1,873.81	0.44	0.66	0.90	855
**PHI[3]**	**Macro-**	16	No	OLS	145.09***	6.89	-5.77	-2.68	0.03	0.17	0.95	13
			Yes	-	-	-	-	-	-	-	-	-
	**Meso-**	79	No	OLS	246.33***	-39.01	86.02	95.49	0.16	0.40	0.86	76
			Yes	SLX	-	-31.63	75.27	89.48	0.14	0.37	0.88	74
	**Micro-**	1,154	No	OLS	3,367.21***	-1,039.38	2,090.75	2,121.06	0.36	0.60	0.92	1,149
			Yes	SDEM	-	-898.42	1,818.84	1,874.40	0.26	0.51	0.94	1,144

[1] logLik: logarithmic Likelihood; AIC: Akaike information criterion; BIC: Bayesian Information Criterion; ML_{unb}: Unbiased Maximum Likelihood estimator of the error variance; SER: Standard error of regression; R2adj: Adjusted coefficient of determination; df: model degrees of freedom.

[2] F-Value. Prob. > F: (***: $<10^{-4}$)(**: $<10^{-2}$)(*: $<10^{-1}$)(n.s.: $>10^{-1}$)

[3] PAN: Pantropical; ZAM: Zambia; ECU: Ecuador; PHI: Philippines.

Table B. Summary of descriptive statistics for all tested explanatory and dependent variables in the twelve samples: all countries aggregated and country-specific across jurisdictional levels (macro- to micro-). N corresponds to the final simple size for each sample, after removing the administrative units from the original sample size of the source dataset, where information for any of the variables was missing.

		**Pantropical (All countries)**			**Zambia**			**Ecuador**			**Philippines**		
		**Macro- N = 49**	**Meso- N = 361**	**Micro- N = 3,035**	**Macro- N=9**	**Meso- N = 70**	**Micro- N = 1,016**	**Macro- N = 24**	**Meso- N = 212**	**Micro- N = 865**	**Macro- N = 16**	**Meso- N = 79**	**Micro- N = 1,154**
**FC** Forest Cover (FA/PVA) [%]	**μ**	35.55%	31.93%	27.73%	42.16%	38.54%	34.64%	41.49%	33.30%	32.01%	22.91%	22.40%	18.45%
	**SD**	22.71%	24.53%	24.87%	13.78%	18.26%	23.31%	27.37%	27.93%	27.68%	12.15%	15.39%	20.81%
	**Min.**	3.73%	0.08%	0.01%	23.27%	7.90%	0.51%	3.73%	0.08%	0.01%	4.35%	1.00%	0.01%
	**Q1**	19.30%	13.60%	6.78%	32.35%	24.33%	14.94%	22.01%	10.15%	8.30%	12.23%	11.84%	2.24%
	**Q2**	29.59%	25.93%	20.99%	41.87%	35.92%	30.34%	31.65%	25.73%	23.64%	23.05%	18.05%	10.13%
	**Q3**	42.80%	46.74%	43.30%	49.36%	50.09%	51.07%	67.85%	51.33%	50.45%	33.33%	32.95%	27.65%
	**Max.**	95.21%	99.47%	99.44%	69.66%	80.91%	92.34%	95.21%	99.47%	99.44%	44.22%	72.02%	97.44%
**A_{TOT}** Total Area [ha x 10^{3}]	**μ**	2,646.24	358.50	37.30	8,367.12	1,075.18	65.82	1,034.15	116.20	24.47	1,846.39	373.69	21.81
	**SD**	3,.356.38	540.49	75.15	4.435.95	800.69	115.01	767.50	191.27	46.44	416.35	247.83	22.84
	**Min.**	78.10	4.00	0.37	2,209.59	73.52	0.39	78.10	4.00	0.37	1,254.99	24.41	0.97
	**Q1**	776.45	51.19	9.10	4.056.90	432.95	17.57	382.12	31.39	6.73	1,499.03	210.07	8.17
	**Q2**	1,738.08	132.54	19.07	8,547.98	975.70	37.66	776.45	60.85	13.37	1,823.08	324.63	14.79
	**Q3**	2,306.25	416.34	40.44	12,635.52	1,562.93	74.72	1,589.20	124.91	27.75	2,071.84	487.87	26.22
	**Max.**	14,751.67	4,029.12	1.629.69	14,751.67	4,029.12	1,629.69	2,962.88	1,992.43	822.53	2,702.13	1,460.79	235.05
**PVA** Potential Veg. Area (PVA/A_{TOT}) [%]	**μ**	91.32%	91.28%	93.62%	97.84%	96.61%	97.09%	87.26%	88.84%	89.03%	93.73%	93.13%	93.99%
	**SD**	8.89%	11.93%	12.46%	3.05%	7.48%	11.09%	10.70%	13.82%	16.54%	3.78%	6.78%	8.30%
	**Min.**	62.41%	34.72%	0.41%	90.33%	61.23%	10.11%	62.41%	34.72%	11.66%	84.20%	54.96%	0.41%
	**Q1**	88.18%	89.26%	93.99%	96.88%	98.04%	99.20%	78.87%	82.01%	84.92%	91.54%	92.64%	92.92%
	**Q2**	94.69%	96.19%	98.12%	99.10%	99.62%	99.90%	89.86%	95.86%	97.11%	94.88%	95.12%	96.23%
	**Q3**	97.27%	99.10%	99.82%	99.71%	99.85%	99.98%	96.77%	98.74%	99.43%	96.47%	96.56%	98.19%
	**Max.**	99.81%	100.00%	100.00%	99.81%	99.96%	100.00%	98.59%	100.00%	100.00%	97.38%	98.59%	100.00%

Table B (continuation)		Pantropical (All countries)			Zambia			Ecuador			Philippines		
		**Macro- N = 49**	**Meso- N = 361**	**Micro- N = 3,035**	**Macro- N=9**	**Meso- N = 70**	**Micro- N = 1,016**	**Macro- N = 24**	**Meso- N = 212**	**Micro- N = 865**	**Macro- N = 16**	**Meso- N = 79**	**Micro- N = 1,154**
**PP_{FA}** Population Pressure (POP/FA) [pers./ha]	**μ**	10.04	104.45	511.99	0.78	1.95	3.14	4.45	155.43	129.50	23.65	58.48	1,246.69
	**SD**	19.05	1,447.54	6,185.59	0.71	4.51	7.23	6.21	1,886.44	1,060.01	28.34	160.30	9,947.92
	**Min.**	0.03	0.00	0.00	0.09	0.04	0.03	0.03	0.00	0.00	2.13	0.51	0.07
	**Q1**	0.77	0.54	0.52	0.25	0.20	0.20	0.30	0.54	0.37	4.73	5.54	5.79
	**Q2**	3.12	2.74	3.14	0.64	0.53	0.64	2.81	2.50	1.79	14.96	15.23	29.06
	**Q3**	10.36	13.41	25.71	1.18	1.65	2.43	5.21	9.97	9.96	34.95	35.67	168.30
	**Max.**	115.67	27,467.46	236,902.13	2.35	27.59	68.16	29.70	27,467.46	18,838.40	115.67	1.265.73	236.902.13
**RD** Road Density [km/km^2]	**μ**	0.54	0.64	0.59	0.13	0.22	0.22	0.56	0.70	0.73	0.72	0.84	0.82
	**SD**	0.41	0.80	0.85	0.12	0.38	0.42	0.39	0.85	1.04	0.41	0.81	0.87
	**Min.**	0.03	0.01	0.00	0.03	0.01	0.00	0.03	0.01	0.00	0.30	0.16	0.00
	**Q1**	0.12	0.16	0.11	0.06	0.05	0.04	0.16	0.21	0.19	0.38	0.38	0.31
	**Q2**	0.46	0.40	0.33	0.08	0.07	0.10	0.62	0.42	0.44	0.57	0.58	0.58
	**Q3**	0.77	0.78	0.77	0.19	0.15	0.23	0.82	0.83	0.87	1.11	1.02	1.06
	**Max.**	1.59	6.24	15.57	0.39	2.21	6.33	1.52	6.24	15.57	1.59	5.44	10.05
**FL** Flatness: Surface with slope under 16% [%]	**μ**	62.59%	61.25%	68.82%	95.62%	96.32%	96.81%	51.77%	50.50%	43.92%	60.24%	59.03%	62.85%
	**SD**	26.85%	32.11%	31.78%	6.22%	5.87%	7.28%	29.02%	33.04%	33.11%	12.17%	18.72%	24.45%
	**Min.**	17.55%	3.46%	1.47%	80.34%	76.43%	40.33%	17.55%	3.46%	1.47%	26.49%	9.71%	4.03%
	**Q1**	39.42%	31.01%	43.13%	94.29%	95.62%	97.85%	23.33%	20.30%	13.96%	55.69%	44.53%	46.14%
	**Q2**	63.05%	63.17%	78.33%	96.77%	99.34%	99.86%	43.61%	45.17%	36.16%	61.87%	61.29%	64.94%
	**Q3**	87.93%	95.25%	99.37%	99.81%	99.92%	100.00%	84.24%	87.95%	71.82%	67.30%	73.09%	83.30%
	**Max.**	100.00%	100.00%	100.00%	100.00%	100.00%	100.00%	96.93%	100.00%	100.00%	77.32%	94.38%	100.00%
**CSI** Crop Suitability Index [%]	**μ**	30.57%	31.70%	35.95%	40.56%	42.36%	47.30%	26.48%	29.15%	28.45%	31.08%	29.12%	31.57%
	**SD**	14.81%	19.24%	22.98%	18.81%	22.19%	25.13%	13.10%	18.57%	21.14%	12.78%	14.64%	17.98%
	**Min.**	2.76%	0.01%	0.02%	8.77%	0.69%	0.04%	2.76%	0.01%	0.02%	12.62%	4.26%	0.04%
	**Q1**	16.78%	16.79%	17.69%	24.27%	24.59%	26.79%	16.30%	14.48%	10.95%	17.80%	18.01%	17.79%
	**Q2**	28.49%	29.07%	32.64%	42.64%	45.54%	47.95%	22.52%	25.37%	22.64%	33.24%	27.98%	30.27%
	**Q3**	42.32%	44.82%	52.41%	57.37%	59.70%	67.36%	34.18%	42.05%	44.02%	40.10%	41.46%	42.95%
	**Max.**	62.99%	84.85%	101.15%	62.99%	82.51%	100.00%	51.98%	84.85%	90.95%	52.27%	69.97%	86.52%

Table B (continuation)		Pantropical (All countries)			Zambia			Ecuador			Philippines		
		Macro- N = 49	Meso- N = 361	Micro- N = 3,035	Macro- N=9	Meso- N = 70	Micro- N = 1,016	Macro- N = 24	Meso- N = 212	Micro- N = 865	Macro- N = 16	Meso- N = 79	Micro- N = 1,154
**CY** Cereal Area Yield [kcal/ha]	**μ**	7,510.06	-	-	7,127.25	-	-	5,946.26	-	-	10,071.09	10,554.85	-
	**SD**	3,177.10	-	-	2,141.76	-	-	3,149.61	-	-	1,909.63	3,773.80	-
	**Min.**	1,686.56	-	-	2,763.21	-	-	1,686.56	-	-	7,720.64	2,466.80	-
	**Q1**	4,949.02	-	-	5,932.17	-	-	3,168.12	-	-	8,777.74	8,371.92	-
	**Q2**	7,882.17	-	-	7,239.70	-	-	5,475.40	-	-	9,730.05	9,928.80	-
	**Q3**	9,757.03	-	-	8,944.78	-	-	8,727.60	-	-	11,354.77	12,378.83	-
	**Max.**	15,152.37	-	-	9,794.32	-	-	12,163.99	-	-	15,152.37	29,402.80	-

Table C. Results of multi-collinearity check: Multivariate Analysis. Correlation between variables at each sample. Grey cells: >0.6.

Variable 1	Variable 2	Pantropical (All countries)			Zambia			Ecuador			Philippines		
		Macro- N = 49	Meso- N = 361	Micro- N = 3.035	Macro- N = 9	Meso- N = 70	Micro- N = 1.016	Macro- N = 24	Meso- N = 212	Micro- N = 865	Macro- N = 16	Meso- N = 79	Micro- N = 1.154
A_{TOT}^	PVA^	0.51	0.27	0.10	0.17	0.24	0.06	0.32	0.13	-0.03	0.31	0.13	0.14
A_{TOT}^	PP_{FA}^	-0.37	-0.38	-0.50	-0.85	-0.80	-0.53	-0.58	-0.55	-0.45	-0.60	-0.27	-0.48
A_{TOT}^	RD^	-0.60	-0.55	-0.60	-0.85	-0.76	-0.58	-0.53	-0.53	-0.48	-0.11	-0.27	-0.42
A_{TOT}^	FL^	0.52	0.37	0.22	0.60	0.06	0.02	0.20	0.07	0.21	-0.28	0.04	-0.28
A_{TOT}^	CSI^	0.39	0.25	0.22	0.76	0.68	0.14	0.24	0.06	0.19	-0.22	0.28	0.08
A_{TOT}^	CY^	0.12	-	-	-0.46	-	-	-0.11	-	-	-0.06	0.03	-
PVA^	PP_{FA}^	-0.21	-0.27	-0.13	-0.15	-0.16	-0.10	-0.37	-0.28	-0.14	-0.43	-0.53	-0.21
PVA^	RD^	-0.40	-0.32	-0.11	0.12	0.09	0.05	-0.43	-0.33	-0.13	-0.47	-0.56	-0.16
PVA^	FL^	0.59	0.19	0.10	-0.14	0.09	-0.01	0.56	0.13	0.10	-0.51	-0.37	-0.16
PVA^	CSI^	0.61	0.33	0.25	0.05	0.17	0.26	0.71	0.32	0.34	0.43	0.36	0.05
PVA^	CY^	0.15	-	-	-0.09	-	-	-0.05	-	-	-0.19	-0.13	-
PP_{FA}^	RD^	0.85	0.74	0.62	0.82	0.73	0.43	0.89	0.69	0.63	0.74	0.79	0.56
PP_{FA}^	FL^	-0.08	0.18	0.12	-0.66	0.04	0.08	-0.12	0.31	0.23	0.65	0.60	0.54
PP_{FA}^	CSI^	-0.15	-0.12	-0.11	-0.42	-0.49	0.00	-0.20	-0.03	-0.19	-0.04	-0.21	0.03
PP_{FA}^	CY^	0.55	-	-	0.40	-	-	0.47	-	-	0.06	-0.04	-
RD^	FL^	-0.37	-0.13	-0.14	-0.49	0.04	0.01	-0.29	0.06	0.07	0.39	0.33	0.37
RD^	CSI^	-0.42	-0.26	-0.28	-0.75	-0.67	-0.20	-0.34	-0.06	-0.22	-0.44	-0.44	-0.19
RD^	CY^	0.40	-	-	0.61	-	-	0.44	-	-	0.26	0.17	-
FL^	CSI^	0.45	0.18	0.25	0.12	-0.14	0.12	0.51	0.14	0.15	0.10	0.09	0.15
FL^	CY^	0.12	-	-	-0.31	-	-	-0.04	-	-	0.08	-0.15	-
CSI^	CY^	-0.10	-	-	-0.51	-	-	-0.17	-	-	-0.30	-0.20	-

Table D. Simple linear regressions for the seven selected explanatory variables in the twelve samples. Coeff.: Coefficient. SE: Standard Error.

		Macro-						Meso-						Micro-					
		**R^2**	**Pr > F**	**Coeff.**	**SE**	**t-Val**	**Pr > |t|**	**R^2**	**Pr > F**	**Coeff.**	**SE**	**t-Val**	**Pr > |t|**	**R^2**	**Pr > F**	**Coeff.**	**SE**	**t-Val**	**Pr > |t|**
**Pantropical (All countries) N = 49 N = 361 N = 3,035**	**A_{TOT}^**	0.08	*	-0.35	0.17	-2.07	*	0.14	***	-0.61	0.082	-7.51	***	0.16	***	-0.81	0.033	-24.25	***
	**Interc**			0.69		4.09	**			1.09		13.33	***			1.58		47.29	***
	**PVA^**	<0.01	n.s.	-0.12	0.18	-0.68	n.s.	0.01	*	-0.20	0.087	-2.28	*	<0.01	*	-0.076	0.037	-2.08	*
	**Interc**			0.69		3.93	*			1.09		12.48	***			1.58		43.31	***
	**PP_{FA}^**	0.78	***	1.07	0.083	12.96	***	0.75	***	1.44	0.044	32.84	***	0.78	***	1.78	0.017	93.49	***
	**Interc**			0.69		8.37	***			1.09		24.79	***			1.58		105.45	***
	**RD^**	0.42	***	0.79	0.14	5.82	***	0.24	***	0.81	0.077	10.59	***	0.19	***	0.88	0.033	26.93	***
	**Interc**			0.69		5.13	***			1.09		14.19	***			1.58		48.18	***
	**FL^**	<0.01	n.s.	-0.029	0.18	-0.16	n.s.	0.28	**	0.28	0.086	3.20	**	0.02	***	0.29	0.036	8.13	***
	**Interc**			0.69		3.92	**			1.09		12.57	***			1.58		43.75	***
	**CSI^**	<0.01	n.s.	0.093	0.18	0.52	n.s.	<0.01	n.s.	0.091	0.088	1.03	n.s.	<0.01	n.s	0.03	0.037	0.83	n.s.
	**Interc**			0.69		3.93	**			1.09		12.41	***			1.58		43.29	***
	**CY^**	0.23	**	0.59	0.16	3.79	**	-	-	-	-	-	-	-	-	-	-	-	-
	**Interc**			0.69		4.47	***			-		-	-			-		-	-
**Zambia N=9 N=70 N =1016**	**A_{TOT}^**	<0.01	n.s.	-0.01	0.21	-0.05	n.s.	0.09	*	-0.26	0.1001	-2.56	*	0.15	***	-0.52	0.039	-13.26	***
	**Interc**			0.33		1.57	n.s.			0.54		5.36	***			0.87		22.28	***
	**PVA^**	0.06	n.s.	-0.14	0.21	-0.66	n.s.	<0.01	n.s.	0.057	0.104	0.54	n.s.	<0.01	*	-0.11	0.042	-2.51	*
	**Interc**			0.33		1.62	n.s.			0.54		5.13	***			0.87		20.63	***
	**PP_{FA}^**	0.23	n.s.	0.27	0.19	1.43	n.s.	0.44	***	0.57	0.079	7.28	***	0.65	***	1.09	0.025	43.83	***
	**Interc**			0.33		1.79	n.s.			0.54		6.83	***			0.87		34.99	***
	**RD^**	<0.01	n.s.	-0.018	0.21	-0.09	n.s.	0.02	n.s.	0.13	0.104	1.29	n.s.	0.05	***	0.31	0.041	7.49	***
	**Interc**			0.33		1.57	n.s.			0.54		5.18	***			0.87		21.13	***
	**FL^**	0.05	n.s.	-0.12	0.21	-0.59	n.s.	<0.01	n.s.	-0.0028	0.105	-0.03	n.s.	0.01	**	0.14	0.042	3.40	**
	**Interc**			0.33		1.61	n.s.			0.54		5.12	***			0.87		20.68	***
	**CSI^**	0.28	n.s.	0.29	0.18	1.64	n.s.	0.02	n.s.	0.10	0.104	1.05	n.s.	0.06	***	0.32	0.042	7.79	***
	**Interc**			0.33		1.85	n.s.			0.54		5.16	***			0.87		21.17	***
	**CY^**	0.06	n.s.	-0.13	0.21	-0.64	n.s.	-	-	-	-	-	-	-	-	-	-	-	-
	**Interc**			0.33		1.62	n.s.			-		-	-			-		-	-

	**Table D**			**Macro-**						**Meso-**						**Micro-**			
	**(continuation)**	**R^2**	**Pr > F**	**Coeff.**	**SE**	**t-Val**	**Pr > |t|**	**R^2**	**Pr > F**	**Coeff.**	**SE**	**t-Val**	**Pr > |t|**	**R^2**	**Pr > F**	**Coeff.**	**SE**	**t-Val**	**Pr > |t|**
**Ecuador - N = 24 N = 212 N = 865**	**A_{TOT}^**	0.41	*	-0.92	0.24	-3.91	**	0.30	***	-1.07	0.11	-9.46	***	0.19	***	-0.90	0.064	-14.09	***
	**Interc**			0.38		1.63	n.s.			1.11		9.76	***			1.26		19.70	***
	**PVA^**	0.05	n.s.	-0.32	0.30	-1.07	n.s.	<0.01	n.s.	-0.20	0.13	-1.51	***	<0.01	n.s	-0.069	0.071	-0.98	n.s.
	**Interc**			0.38		1.28	n.s.			1.11		8.22	***			1.26		17.77	***
	**PP_{FA}^**	0.89	***	1.36	0.10	13.34	***	0.84	***	1.80	0.054	33.16	***	0.84	***	1.91	0.029	66.35	***
	**Interc**			0.38		3.78	**			1.11		20.41	***			1.26		43.88	***
	**RD^**	0.63	***	1.14	0.19	6.06	***	0.27	***	1.02	0.12	8.83	***	0.24	***	1.014	0.062	16.34	***
	**Interc**			0.38		2.05	n.s.			1.11		9.57	***			1.26		20.33	***
	**FL^**	0.02	n.s.	-0.20	0.30	-0.64	n.s.	0.05	**	0.45	0.13	3.43	***	0.02	***	0.29	0.071	4.17	***
	**Interc**			0.38		1.26	n.s.			1.11		8.40	**			1.26		17.94	***
	**CSI^**	<0.01	n.s.	-0.03	0.31	-0.09	n.s.	<0.01	n.s.	1.11	0.13	1.25	***	<0.01	*	-0.18	0.071	-2.55	*
	**Interc**			0.38		1.25	n.s.			0.17		8.20	n.s.			1.26		17.83	***
	**CY^**	0.24	*	0.70	0.27	2.62	*	-	-	-	-	-	-	-	-	-	-	-	-
	**Interc**			0.38		1.43	n.s.			-		-	-			-		-	-
**Philippines - N = 16 N = 79 N = 1.154**	**A_{TOT}^**	0.31	*	-0.43	0.17	-2.53	*	0.03	n.s.	-0.19	0.12	-1.58	n.s.	0.11	***	-0.70	0.059	-11.88	***
	**Interc**			1.36		8.10	***			1.52		12.51	***			2.44		41.38	***
	**PVA^**	<0.01	n.s.	-0.064	0.20	-0.32	n.s.	0.08	*	-0.30	0.12	-2.54	*	0.06	n.s	-0.054	0.063	-0.86	n.s.
	**Interc**			1.36		6.73	***			1.52		12.82	***			2.44		39.07	***
	**PP_{FA}^**	0.84	***	0.69	0.082	8.47	***	0.81	***	0.98	0.054	18.18	***	0.87	***	1.98	0.023	85.89	***
	**Interc**			1.36		16.59	***			1.52		28.33	***			2.44		106.28	***
	**RD^**	0.32	*	0.43	0.17	2.58	*	0.38	***	0.67	0.098	6.81	***	0.16	***	0.86	0.057	15.00	***
	**Interc**			1.36		8.14	***			1.52		15.59	***			2.44		42.70	***
	**FL^**	0.25	*	0.39	0.18	2.24	*	0.31	***	0.60	0.103	5.83	***	0.23	***	1.01	0.055	18.36	***
	**Interc**			1.36		7.82	***			1.52		14.79	***			2.44		44.41	***
	**CSI^**	0.01	n.s.	0.082	0.20	0.41	n.s.	<0.01	n.s.	-0.05	0.12	-0.41	n.s.	<0.01	**	0.18	0.062	2.96	**
	**Interc**			1.36		6.74	***			1.52		12.33	***			2.44		39.21	***
	**CY^**	<0.01	n.s.	-0.029	0.20	-0.15	n.s.	<0.01	n.s.	-0.08	0.12	-0.66	n.s.	-	-	-	-	-	-
	**Interc**			1.36		6.71	***			1.52		12.35	***			-		-	-

Table E. Impacts for all **macro-level** samples in OLS models. Coef: Coefficient. SE: Standard Error. (^: linearized and standardized variable) (X: variable eliminated by de model) (XX: not applicable in this model) (-: Collinearity > 0.6). (***: $<10^{-4}$) (**: $<10^{-2}$) (*: $<10^{-1}$) (ns: $>10^{-1}$).

		OLS			
		**Coef**	**SE**	**T-val**	**P> |t|**
**Zambia - N = 9**	**Inter**	0.33	0.08	4.25	*
	**A_{TOT}^**	-	-	-	-
	**PVA^**	-0.10	0.08	-1.21	n.s.
	**PP_{FA}^**	0.48	0.09	5.40	**
	**RD^**	-	-	-	-
	**FL^**	-	-	-	-
	**CSI^**	0.45	0.10	4.76	**
	**CY^**	-0.10	0.09	-1.11	n.s.
**Ecuador - N = 24**	**Inter**	0.38	0.06	6.02	***
	**A_{TOT}^**	-0.28	0.08	-3.39	**
	**PVA^**	-	-	-	-
	**PP_{FA}^**	1.17	0.09	12.84	***
	**RD^**	-	-	-	-
	**FL^**	-0.20	0.08	-2.72	*
	**CSI^**	0.41	0.08	5.36	***
	**CY^**	0.18	0.08	2.46	*
**Philippines – N= 16**	**Inter**	1.36	0.44	31.20	***
	**A_{TOT}^**	x	x	x	x
	**PVA^**	0.29	0.05	6.04	***
	**PP_{FA}^**	0.82	0.05	16.97	***
	**RD^**	-	-	-	-
	**FL^**	-	-	-	-
	**CSI^**	x	x	x	x
	**CY^**	x	x	x	x
**Pantropical – N= 49**	**Inter**	0.69	0.75	9.30	***
	**A_{TOT}^**	x	x	x	x
	**PVA^**	-	-	-	-
	**PP_{FA}^**	1.11	0.76	14.75	***
	**RD^**	-	-	-	-
	**FL^**	x	x	x	x
	**CSI^**	0.26	0.08	3.46	**
	**CY^**	x	x	x	x

Table F. Impacts for all samples at **meso-level** in both OLS and spatial models. Coef: Coefficient. SE: Standard Error. (^: linearized and standardized variable) (***: <10-4) (**: <10-2) (*: <10-1) (n.s.: >10-1) (X: variable eliminated by de model) (XX: not applicable in this model) (-: Collinearity > 0.6).

		OLS				Spatial Model: SDEM, SEM, or SLX											
		Coef	SE	t-Val	P>|t|	Coef	SE	z-Val	P>|z|	Indirect impacts (lag X) (θ neighbors)				Total impacts			
**Zambia - N = 70**	λ(Err)	xx	xx	xx	xx	0.43	0.13	3.23	**	Coef	SE	z-Val	P>|z|	Coef	SE	z-Val	P>|z|
	Inter	0.53	0.06	9.37	***	0.55	0.09	5.99	***								
	A_{TOT}^	-	-	-	-	-	-	-	-	xx	xx	xx	xx	xx	xx	xx	xx
	PVA^	-	-	-	-	-	-	-	-	xx	xx	xx	xx	xx	xx	xx	xx
	PP_{FA}^	0.82	0.07	12.54	***	0.81	0.07	11.63	***	xx	xx	xx	xx	xx	xx	xx	xx
	RD^	-	-	-	-	-	-	-	-	xx	xx	xx	xx	xx	xx	xx	xx
	FL^	x	x	x	x	X	x	x	x	xx	xx	xx	xx	xx	xx	xx	xx
	CSI^	0.51	0.07	7.81	***	0.49	0.08	6.28	***	xx	xx	xx	xx	xx	xx	xx	xx
	CY^	xx	xx	xx	xx	Xx	xx	xx	xx	xx	xx	xx	xx	xx	xx	xx	xx
**Ecuador - N = 212**	λ(Err)	xx	xx	xx	xx	0.52	0.07	7.46	***	Coef	SE	z-Val	P>|z|	Coef	SE	z-Val	P>|z|
	Inter	1.11	0.05	23.4	***	1.11	0.08	13.16	***								
	A_{TOT}^	x	x	x	x	x	x	x	x	x	x	x	x	x	x	x	x
	PVA^	0.33	0.05	6.14	***	0.27	0.05	4.99	***	0.14	0.11	1.30	n.s.	0.41	0.12	3.52	**
	PP_{FA}^	1.97	0.05	37.1	***	1.85	0.06	28.83	***	0.21	0.10	2.01	*	2.06	0.10	19.69	***
	RD^	-	-	-	-	-	-	-	-	-	-	-	-	-	-	-	-
	FL^	-0.23	0.05	-4.43	***	0.02	0.08	0.20	n.s	-0.37	0.12	-3.15	**	-0.35	0.10	-3.51	**
	CSI^	0.15	0.05	2.87	**	0.16	0.05	3.25	**	-0.02	0.10	-0.21	n.s.	0.14	0.11	1.26	n.s.
	CY^	xx	xx	xx	xx	xx	xx	xx	xx	xx	xx	xx	Xx	xx	xx	xx	xx
**Philippines - N = 79**	λ(Err)	xx	xx	xx	xx	xx	xx	xx	xx	Coef	SE	z-Val	P>|z|	Coef	SE	z-Val	P>|z|
	Inter	1.52	0.04	33.5	***	1.52	0.04	36.26	***								
	A_{TOT}^	x	x	x	x	x	x	x	x	x	x	x	x	x	x	x	x
	PVA^	0.30	0.05	5.61	***	0.22	0.05	4.04	**	0.27	0.09	3.15	**	0.49	0.08	6.09	***
	PP_{FA}^	1.14	0.05	21.2	***	1.19	0.06	20.14	***	-0.04	0.09	-0.45	n.s.	1.15	0.08	15.43	***
	RD^	-	-	-	-	-	-	-	-	-	-	-	-	-	-	-	-
	FL^	x	x	x	x	x	x	x	x	x	x	x	x	x	x	x	x
	CSI^	x	x	x	x	x	x	x	x	x	x	x	x	x	x	x	x
	CY^	x	x	x	x	x	x	x	x	x	x	x	x	x	x	x	x
**Pantropical – N = 361**	λ(Err)	xx	xx	xx	xx	0.72	0.04	18.99	***	Coef	SE	z-Val	P>|z|	Coef	SE	z-Val	P>|z|
	Inter	1.09	0.04	26.85	***	1.08	0.10	10.46	***								
	A_{TOT}^	-0.16	0.05	-3.57	**	-0.09	0.05	-1.87	*	xx	xx	xx	xx	xx	xx	xx	xx
	PVA^	0.15	0.04	3.32	**	0.19	0.04	4.76	***	xx	xx	xx	xx	xx	xx	xx	xx
	PP_{FA}^	1.45	0.05	32.49	***	1.636	0.05	31.90	***	xx	xx	xx	xx	xx	xx	xx	xx
	RD^	-	-	-	-	-	-	-	-	xx	xx	xx	xx	xx	xx	xx	xx
	FL^	x	x	x	x	x	x	x	x	xx	xx	xx	xx	xx	xx	xx	xx
	CSI^	0.26	0.04	5.98	***	0.21	0.04	5.37	***	xx	xx	xx	xx	xx	xx	xx	xx
	CY^	xx	xx	xx	xx	xx	xx	xx	xx	xx	xx	xx	xx	xx	xx	xx	xx

Table G. Impacts for all **micro-level** samples in OLS models. Coef: Coefficient. SE: Standard Error. (^: linearized and standardized variable) (X: variable eliminated by de model) (XX: not applicable in this model) (-: Collinearity > 0.6). (***: <10^{-4}) (**: <10^{-2}) (*: <10^{-1}) (n.s.: >10^{-1}).

		OLS			
		**Coef**	**SE**	**T-val**	**P> |t|**
**Zambia – N = 1,016**	**Inter**	0.87	0.02	38.86	***
	**A_{TOT}^**	x	x	x	x
	**PVA^**	-0.07	0.02	-3.11	**
	**PP_{FA}^**	1.13	0.03	44.77	***
	**RD^**	-0.11	0.03	-4.17	***
	**FL^**	x	x	x	x
	**CSI^**	0.32	0.02	13.36	***
	**CY^**	xx	xx	xx	xx
**Ecuador – N = 865**	**Inter**	1.26	0.03	47.40	***
	**A_{TOT}^**	x	x	x	x
	**PVA^**	0.17	0.03	6.08	***
	**PP_{FA}^**	2.02	0.03	71.34	***
	**RD^**	-	-	-	-
	**FL^**	-0.22	0.03	-8.00	***
	**CSI^**	0.19	0.03	6.48	***
	**CY^**	xx	xx	xx	xx
**Philippines – N = 1,154**	**Inter**	2.45	0.02	139.14	***
	**A_{TOT}^**	0.26	0.02	12.76	***
	**PVA^**	0.35	0.02	19.48	***
	**PP_{FA}^**	2.32	0.02	102.84	***
	**RD^**	-0.27	0.02	-12.34	***
	**FL^**	x	x	x	x
	**CSI^**	x	x	x	x
	**CY^**	xx	xx	xx	xx
**Pantropical – N= 3,035**	**Inter**	1.58	0.02	97.25	***
	**A_{TOT}^**	0.06	0.02	3.00	***
	**PVA^**	0.11	0.02	6.24	***
	**PP_{FA}^**	1.85	0.02	98.07	***
	**RD^**	-	-	-	-
	**FL^**	x	x	x	x
	**CSI^**	0.20	0.02	11.63	***
	**CY^**	xx	xx	xx	xx
