# Supplementary material for: Scale and context dependency of deforestation drivers: Insights from spatial econometrics in the tropics
Source: PLoS One. 2020 Jan 29;15(1):e0226830. doi: 10.1371/journal.pone.0226830 (PMC6988916; doi:10.1371/journal.pone.0226830)
Supplement: S4 File — (PDF) [file pone.0226830.s005.pdf]

Executive summary of the main findings

- We could provide a first set of highly significant econometric models of pantropical deforestation that consider subnational administrative units.
- We demonstrated that neglecting spatial dependencies in this type of studies could lead to several misinterpretations.
- We identified recurrent drivers across countries and scales, namely population pressure and the natural condition of land suitability for crop production.
- The impacts of demography on forest cover were strikingly strong across contexts, suggesting clear limitations of sectoral policy.
- Our findings revealed scale and context dependencies, such as an increased heterogeneity at local scopes, with a larger and more diverse number of significant determinants of forest cover.
- We detected stronger spatial interactions at smaller levels, providing empirical evidence that certain deforestation forces occur independently of the existing *de jure* governance boundaries. This suggests the need for jurisdictional approaches that acknowledge the local heterogeneity of landscapes in research and policy implementation.
